# Modulation of Closed−State Inactivation in Kv2.1/Kv6.4 Heterotetramers as Mechanism for 4−AP Induced Potentiation

**DOI:** 10.1371/journal.pone.0141349

**Published:** 2015-10-27

**Authors:** Jeroen I. Stas, Elke Bocksteins, Alain J. Labro, Dirk J. Snyders

**Affiliations:** Laboratory for Molecular Biophysics, Physiology and Pharmacology, Department of Biomedical Sciences, University of Antwerp, CDE, Universiteitsplein 1, Antwerp, Belgium; Indiana University School of Medicine, UNITED STATES

## Abstract

The voltage−gated K^+^ (Kv) channel subunits Kv2.1 and Kv2.2 are expressed in almost every tissue. The diversity of Kv2 current is increased by interacting with the electrically silent Kv (KvS) subunits Kv5−Kv6 and Kv8−Kv9, into functional heterotetrameric Kv2/KvS channels. These Kv2/KvS channels possess unique biophysical properties and display a more tissue-specific expression pattern, making them more desirable pharmacological and therapeutic targets. However, little is known about the pharmacological properties of these heterotetrameric complexes. We demonstrate that Kv5.1, Kv8.1 and Kv9.3 currents were inhibited differently by the channel blocker 4−aminopyridine (4−AP) compared to Kv2.1 homotetramers. In contrast, Kv6.4 currents were potentiated by 4−AP while displaying moderately increased affinities for the channel pore blockers quinidine and flecainide. We found that the 4−AP induced potentiation of Kv6.4 currents was caused by modulation of the Kv6.4−mediated closed−state inactivation: suppression by 4−AP of the Kv2.1/Kv6.4 closed−state inactivation recovered a population of Kv2.1/Kv6.4 channels that was inactivated at resting conditions, i.e. at a holding potential of −80 mV. This modulation also resulted in a slower initiation and faster recovery from closed−state inactivation. Using chimeric substitutions between Kv6.4 and Kv9.3 subunits, we demonstrated that the lower half of the S6 domain (S6c) plays a crucial role in the 4−AP induced potentiation. These results demonstrate that KvS subunits modify the pharmacological response of Kv2 subunits when assembled in heterotetramers and illustrate the potential of KvS subunits to provide unique pharmacological properties to the heterotetramers, as is the case for 4−AP on Kv2.1/Kv6.4 channels.

## Introduction

**Fig 1:**
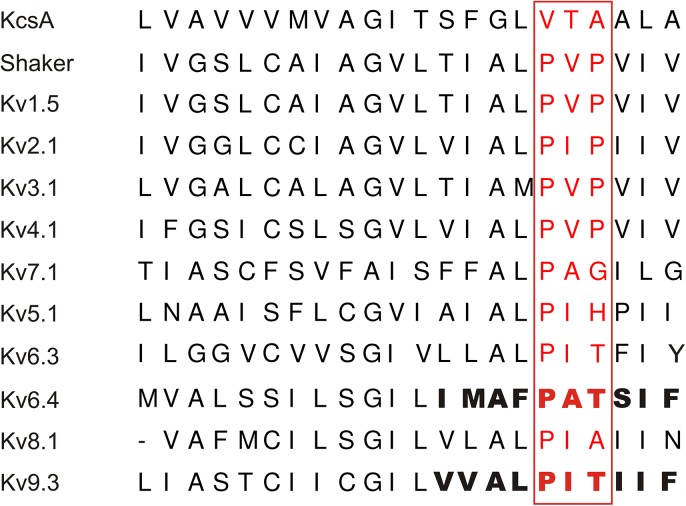
Sequence alignment of the S6 segment of various Kv and KvS subunits. The PXP motif (red) is conserved between the *Shaker* and Kv1−Kv4 subunits. The Kv7.1 subunit displays a glycine (could confer flexibility) while the Kv5, Kv6, Kv8 and Kv9 (or KvS) subunits contain residues that would promote an alpha−helical configuration. The S6c segment used to make the chimeric substitution between Kv6.4 and Kv9.3 is highlighted in bold.

Voltage−gated K^+^ (Kv) channels are K^+^ selective membrane proteins that open, close and/or inactivate in response to changes in the membrane potential [1]. They exist as tetramers of α−subunits each consisting of six transmembrane segments (S1−S6) [2]. The four S5−S6 segments generate the central ion conducting pore while the S1−S4 segments form the voltage−sensing domains (VSDs). The channel pore can be sealed off by the channel gate at the level of the S6 bundle crossing (BC) [3,4]. Opening and closure of this BC gate is controlled by the VSDs that detect changes in the membrane potential. The highly conserved PXP motif (Fig 1) near the intracellular end of S6 (S6c) functions as a hinge that allows the necessary swiveling and bending motions [5–7]. In addition to forming the BC gate, the S6c region (including the PXP motif) has also been implicated in determining the channel’s affinity for pore blockers [8–12].

Based on sequence homology, the Kv superfamily has been divided into eight closely *Shaker*−related subfamilies: Kv1−Kv6 and Kv8−Kv9 [13]. Unlike the Kv1−Kv4 subunits, members of the Kv5, Kv6, Kv8 and Kv9 subfamilies–designated silent Kv (KvS) subunits–do not produce functional homotetramers on their own, despite possessing most of the hallmarks of a typical Kv channel subunit. However, KvS subunits lack the 2^nd^ proline residue of the highly conserved PXP motif. KvS subunits assemble with Kv2 subunits into functional Kv2/KvS heterotetramers that possess unique biophysical properties; KvS subunits shift the voltage dependence of inactivation and/or activation, affect the current density and modulate the kinetics of both activation and deactivation compared to Kv2 homotetramers [14]. The different Kv2/KvS channels also display a more tissue−specific expression compared to Kv2.1 homotetramers which are expressed in almost every tissue, e.g. Kv6.4 is abundantly expressed in brain tissue and weakly expressed in liver, small intestine and colon [15]. Therefore, heterotetramerization of KvS with Kv2 subunits is thought to increase the functional diversity and most likely serves tissue−specific functions for these Kv2/KvS channel complexes. Indeed, heterotetrameric Kv2.1/Kv9.3 channels are involved in hypoxic pulmonary artery vasoconstriction [16,17], mutations in Kv8.2 have been associated with an inherited retinal dystrophy [18] and epilepsy susceptibility [19] and recently the involvement of Kv6.4 in a fast motor neuron biophysical signature has been demonstrated [20].

Because the S6c sequence of KvS subunits differs from that of Kv2 subunits and since certain Kv2/KvS combinations appear to display a more tissue−specific expression than the more ubiquitously expressed Kv2 subunits, these Kv2/KvS heterotetramers have a therapeutic potential as more specific pharmacological targets. In this study, we demonstrate that Kv6.4 subunits confer a distinct pharmacological profile to the Kv2.1/Kv6.4 heterotetramers for the common channel inhibitor 4−aminopyridine (4−AP) in which the Kv6.4 S6c sequence plays an important role. 4−AP is known to inhibit Kv2.1 channels, but surprisingly it potentiated the Kv2.1/Kv6.4 current. This 4−AP response was unique for the Kv2.1/Kv6.4 heterotetramers and not observed with other KvS subunits suggesting that various Kv2.1/KvS heterotetramers harbor a unique pharmacological profile.

## Materials and Methods

### Molecular biology

Human Kv2.1, Kv5.1, Kv6.4, Kv8.1 and mouse Kv9.3 were subcloned in the eGFP−N1 vector (Clontech, Palo Alto, CA, USA) as previously described [15]. The chimeric Kv6.4 and Kv9.3 constructs Kv6.4_S6c_Kv9.3 and Kv9.3_S6c_Kv6.4 in which the C−terminal end of the Kv6.4 and Kv9.3 S6 segments (S6c) were exchanged for that of Kv9.3 and Kv6.4, respectively (Fig 1), were constructed using a loop-in PCR strategy using mutant primers and the Quickchange Site−Directed Mutagenesis kit (Stratagene, La Jolla, CA, USA) as previously reported [21,22]. To confirm the presence of the desired S6c sequence exchange and the absence of unwanted mutations, the amplified cDNA was sequenced.

### Transient transfection and cell culture

Mouse fibroblast Ltk^−^ cells (American Type Culture Collection CLL. 1.3) were chosen for the electrophysiological experiments because of low levels of endogenous current [23,24]. Ltk^-^ cells were cultured in Dulbecco’s modified Eagle’s medium supplemented with 10% horse serum (purchased from Gibco®, Life Technologies, Gent, Belgium) and 1% penicillin/streptomycin at 37°C and under 5% CO_2_. Ltk^−^ cells were transiently transfected with 0.05 μg– 5 μg cDNA of wild type (WT) or mutant channel subunits along with 0.5 μg eGFP as transfection marker using the polyethylenimine reagent (Sigma−Aldrich, St. Louis, MO, USA) according to the manufacturer’s instruction. To ensure that only a population of heterotetrameric Kv2.1/KvS channels was formed, the KvS and Kv2.1 subunits were co−transfected in a 10:1 cDNA ratio. Twenty−four hours post transfection the Ltk^−^ cells were trypsinized and used for the electrophysiological experiments.

### Electrophysiology

Current recordings were obtained in the whole cell configuration at room temperature (20−23°C) with an Axopatch−200B amplifier (Axon Instruments, Union City, CA, USA). The current recordings were low−pass filtered through a Bessel filter and sampled at 1 − 10 kHz with a Digidata 1200A data acquisition system (Axon Instruments). Data storage and command voltages were controlled with the pClamp10 software (Axon Instruments). Pipettes were pulled using a P−2000 puller (Sutter Instruments, Novato, CA, USA) from borosilicate glass capillaries (World Precision Instruments, Sarasota, FL, USA) and afterwards heat polished to obtain patch pipettes with a resistance between 1 MΩ− 2.5 MΩ. The patch pipettes were filled with an intracellular solution (ICS) containing 110 mM KCl, 5 mM K_2_ATP, 5 mM K_4_BAPTA, 2 mM MgCl_2_, 10 mM HEPES and adjusted to pH 7.2 with KOH. The Ltk^−^ cells were continuously perfused with an extracellular solution (ECS) containing 145 mM NaCl, 4 mM KCl, 1 mM MgCl_2_, 1.8 mM CaCl_2_, 10 mM HEPES, 10 mM glucose and adjusted to pH 7.35 with NaOH. Junction potentials between the ICS and ECS were zeroed with the filled pipette in the bath solution. 4−aminopyridine (4−AP) (Sigma−Aldrich), flecainide (S.A. Meda Pharma N.V., Brussels, Belgium) and quinidine (Eli Lilly & co, Indianapolis, IN, USA) were diluted in ECS solution to obtain a stock solution of 180 mM, 2 mM and 1 mM, respectively. Stock solutions were balanced to a pH of 7.35. The desired working concentrations of 4−AP, flecainide and quinidine were made by diluting these stock solutions in ECS solution and were applied by using a fast perfusion system (ALA Scientific Instruments, Farmingdale, NY, USA).

### Data analysis and statistics

Voltage protocols are represented in the figures and were adjusted to the biophysical properties of the different Kv channels. The voltage dependence of activation and inactivation was fitted with a single Boltzmann function according to y = 1 / (1 + exp (−(V–V_1/2_) / k)), with *V* representing the voltage applied, *V*_*1/2*_ the voltage at which 50% of the channels are activated or inactivated, and *k* the slope factor. Time constants of activation (from + 80 to −10 mV) and deactivation (−20 to −70 mV) were obtained by fitting the raw current traces of either the activation or deactivation (not shown) protocol with a single or double exponential function. Dose−response curves were obtained by plotting y, the fraction of current remaining at +30mV, as a function of drug concentration, [D], and fitted with the Hill equation: 1 –y = 1 / (1 + (IC_50_ / [D])^n^_H_), where IC_50_ is the concentration that generates 50% inhibition and n_H_ the Hill coefficient. Results are expressed as mean ± S.E.M. Statistical difference was determined with standard t−test if applicable or the Mann−Whitney Rank Sum test. P < 0.05 was considered significant.

### Nomenclature

We reported in 2002 the cloning and properties of three KvS subunits designated Kv6.3, Kv10.1 and Kv11.1, based on the degree of sequence homology in the S1-S6 region [15]. The Kv subunit nomenclature was revised by IUPHAR in 2005 [13]. Kv10.1 became Kv6.3, Kv6.3 was renamed to Kv6.4 and Kv11.1 to Kv8.2. Thus, the Kv6.4 mentioned in this paper is the KvS subunit that displayed the largest hyperpolarizing shift in the voltage-dependence of inactivation in the original report [15].

## Results

### 4−AP potentiates Kv2.1/Kv6.4 currents while inhibiting other Kv2/KvS combinations

**Fig 2:**
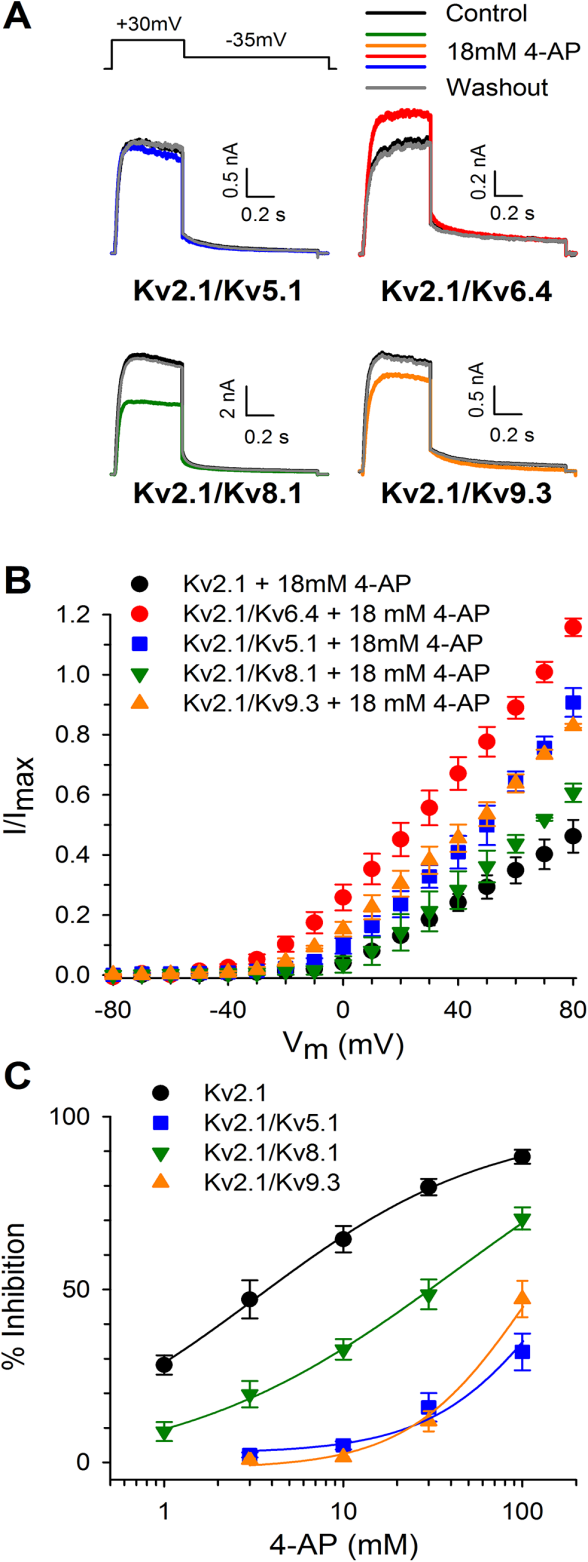
KvS subunits have a distinct pharmacological profile for the channel blocker 4−AP. (A) Effect of 4−AP on Kv2.1/KvS heterotetramers. Application of 4−AP (colored traces) yielded different amounts of block for Kv5.1, Kv8.1 and Kv9.3 containing Kv2.1/KvS complexes. Remarkably, 4−AP caused a current increase for Kv2.1/Kv6.4 heterotetramers. Control traces are shown in black and recorded at +30mV. Washout traces are shown in dark grey. (B) Current−voltage relationships for the different Kv2.1/KvS heterotetramers obtained by plotting the normalized current amplitude (to the current amplitude under control conditions) in function of the pulse potential. (C) Concentration−effect relationships of homotetrameric Kv2.1 (black circles) and heterotetrameric Kv2.1/Kv5.1 (blue squares), Kv2.1/Kv8.1 (green downward triangles) and Kv2.1/Kv9.3 (orange upward triangles) channels. All Kv2.1/KvS heterotetramers possess a reduced 4−AP affinity observed as right−shifted concentration−effect curves. Lines represent the fit of the concentration−effect curves with the Hill equation as mentioned in the material and methods section.

A common feature of all the KvS subunits is that they lack the 2^nd^ proline residue of the PXP motif within the bottom end of the S6 segment (S6c) (Fig 1), a channel region that has been shown to be involved in the interaction with pore blockers [8–10]. Substituting the 2^nd^ proline of the PXP motif by an alanine or glycine drastically reduced the 4−AP affinity of the *Shaker* Kv channel [25]. This raised the question whether the KvS subunits also affect the pharmacological profile of Kv2 containing heteromers, in addition to altering the biophysical properties. Since the effect of 4−AP on Kv2/KvS heteromeric channels has not been investigated extensively, we screened heterotetramers of Kv2.1 and either Kv5.1, Kv6.4, Kv8.1 or Kv9.3 for their sensitivity to 4−AP (Fig 2). These KvS subunits were chosen as representative members for each of the four KvS subfamilies. Application of 18 mM 4−AP, which is the reported IC_50_ value for Kv2.1 inhibition [26,27], blocked 10 ± 2%, 56 ± 2% and 12 ± 1% of Kv5.1, Kv8.1 and Kv9.3 containing currents, respectively (n = 7) (Fig 2A and 2B). Full dose−response relationships for the different heterotetrameric Kv2.1/KvS and for homotetrameric Kv2.1 channels were determined and compared to obtain a relative measurement of the 4−AP sensitivity (Fig 2C). No proper IC_50_ value and Hill coefficient (n_H_) could be determined for the Kv2.1/Kv5.1 and Kv2.1/Kv9.3 channel due to their weak sensitivity for 4-AP inhibition (Fig 2C). Kv2.1/Kv8.1 channels displayed sufficient sensitivity and were inhibited with an IC_50_ of 24.1 ± 2.0 mM and n_H_ = 1.4 ± 0.2 (n = 5). Homotetrameric Kv2.1 channels displayed an IC_50_ of 4.5 ± 1.8 mM (n = 6) for inhibition by 4−AP, and Hill coefficient n_H_ of 1.0 ± 0.1 (n = 6), which is a higher affinity compared to the value of 18mM reported previously [27]. However, this difference most likely reflects the difference in experimental conditions namely the used heterologous expression system. Our IC_50_ value corresponds well with the reported sensitivity in HEK cells and cultivated neurons/myocytes (0.5mM to 5mM) [28,29] but is lower compared to similar experiments performed in oocytes (18mM) [27]. The dose−response relationships of Kv5.1, Kv8.1 and Kv9.3 currents were all right−shifted towards a higher concentration range in comparison to Kv2.1 currents. Thus, Kv5.1, Kv8.1 and Kv9.3 heterotetramers display an altered sensitivity for 4−AP.

Interestingly, application of 4−AP increased the Kv6.4 current by 16 ± 1% (n = 9). To exclude that this effect was due to a potential change in intracellular pH caused by the high 4−AP concentration, we increased the buffering capacity of the “intracellular” pipette solution from 10 mM to 20 mM HEPES; even with this solution we observed an increase in Kv2.1/Kv6.4 currents after 4−AP application. This potentiating effect of 4-AP was not conserved within the Kv6 subfamily because heterotetrameric Kv2.1/Kv6.3 channels (before the IUPHAR classification also known as Kv10.1) were inhibited by 4-AP (S1A Fig), although with a reduced sensitivity compared to homotetrameric Kv2.1 channels (S1 Fig)[13,15].

To examine whether this effect was unique to 4−AP, we tested the effect of two other channel blockers − quinidine and flecainide − on Kv2.1/Kv6.4 channels (S2 Fig). Unlike 4−AP, both quinidine and flecainide blocked Kv2.1/Kv6.4 currents; the IC_50_ values of quinidine and flecainide for heterotetrameric Kv2.1/Kv6.4 channels were 3.0 ± 0.1 μM (n = 7, S2B Fig) and 2.0 ± 0.1 μM (n = 5, S2D Fig), respectively, which is twice as sensitive compared to Kv2.1 homotetramers (IC_50_ = 7.2 ± 0.8 μM, n = 7 and 7.4 ± 0.8 μM, n = 4 respectively). While not extensive, these changes in IC_50_ values were statistically significant (p = 0.008 and p < 0.001 for flecainide and quinidine, respectively).

### 4−AP affects closed−state inactivation in Kv6.4 containing channels

**Fig 3:**
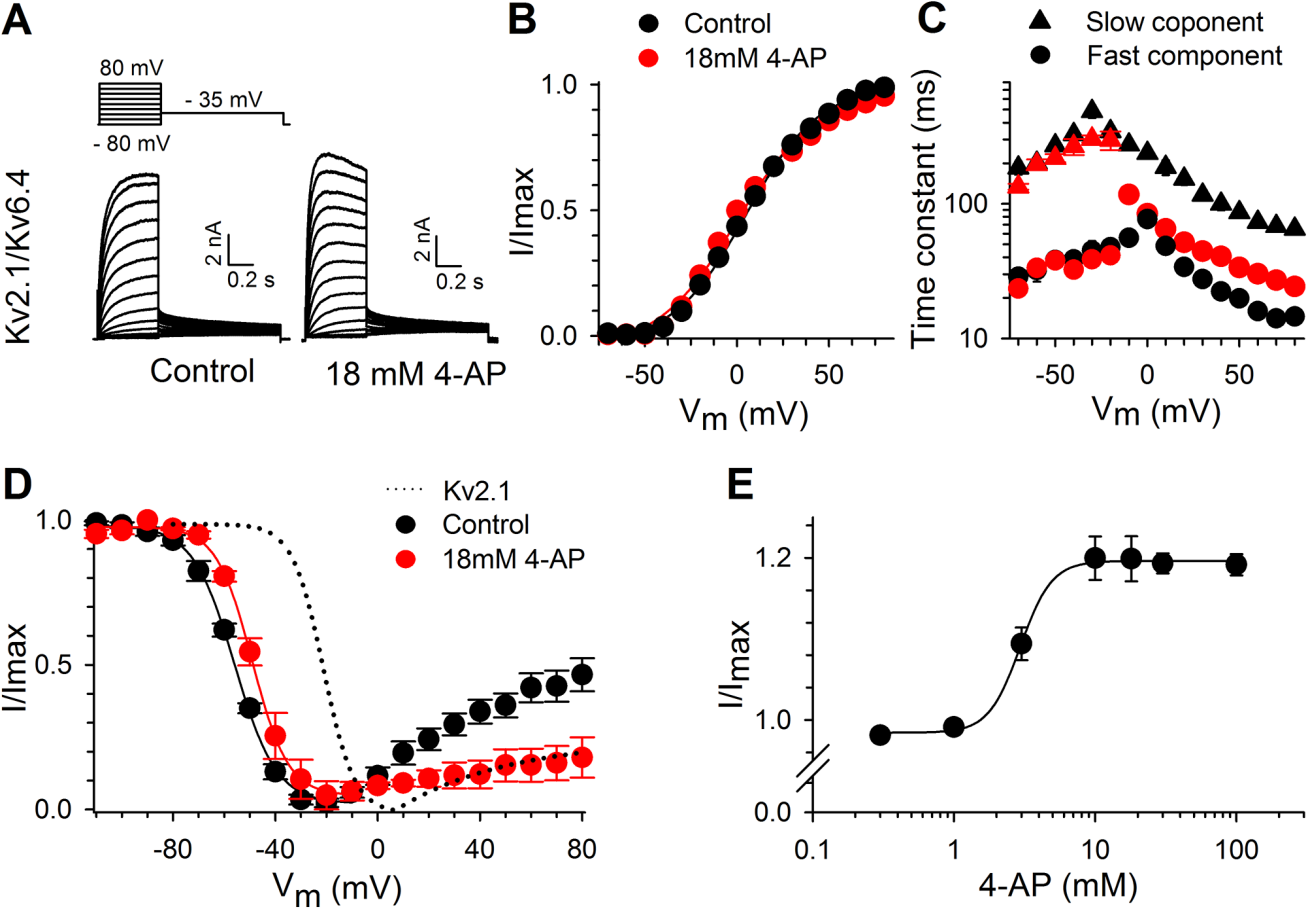
Effect of 4−AP on the gating properties of Kv2.1/Kv6.4 heterotetramers. **(**A) Representative current recordings of Kv2.1/Kv6.4 before (left) and after application of 4−AP (right). 4−AP potentiated Kv2.1/Kv6.4 currents and accelerated the activation kinetics. (B) Voltage dependence of activation with (red) and without 4−AP (black). The voltage dependence of activation was derived from plotting the normalized tail currents in panel A in function of the prepulse potential. (C) Activation and deactivation kinetics of the Kv2.1/Kv6.4 heterotetramers. Time constants were obtained with a double exponential function yielding a fast (circles) and slow (triangles) component under control conditions. 4−AP (red) abolished the slow component in the activation but left the fast component largely unaffected. (D) The voltage dependence of inactivation before (black) and after application of 4−AP (red). The voltage−dependence of inactivation was obtained by plotting the normalized peak amplitude at +60mV after a 5s prepulse in function of the prepulse potential. 4−AP (red symbols) did not significantly alter the voltage dependence of activation but significantly reduced the degree of U−shaped inactivation. For comparison, homomeric Kv2.1 (dotted line) before 4−AP application is included. (E) Concentration−dependence of the 4−AP induced potentiation relative to control. The solid line represents a fit with the Hill equation with EC_50_ of 3.2 ± 0.2 mM (n = 4).

4−AP not only decreased the ionic current of homotetrameric Kv2.1 channels, but also slowed the activation kinetics. This is illustrated in S3A and S3C Fig [30]. Furthermore, we observed that 18 mM 4−AP shifted the voltage dependence of channel activation by approximately 17 mV towards more positive potentials (S3B Fig) and markedly slowed the apparent rate of activation (S3C Fig). In contrast, Fig 3B and Table 1 show that 4−AP did not significantly alter the voltage dependence of activation of heterotetrameric Kv2.1/Kv6.4 channels (p = 0.090). 4−AP did alter the activation time course of Kv2.1/Kv6.4 channels (Fig 3A) but in a different manner compared to Kv2.1 homotetramers (S3C Fig). 4−AP eliminated the slow component of the Kv2.1/Kv6.4 activation time course and although it slowed the fast component 2−fold (Fig 3C), the final result was a relatively faster activation, as observed in Fig 3A.

**Table 1:** Overview of the biophysical properties for Kv2.1, Kv2.1/Kv6.4, Kv2.1/Kv6.4_S6c_Kv9.3 and Kv2.1/Kv9.3_S6c_Kv6.4 channels.

		Control			18mM 4-AP		
		V_1/2_ (mV)	k	n	V_1/2_ (mV)	k	n
Activation	Kv2.1	−2.0 ± 0.9	9.7 ± 0.5	6	**15.4 ± 1.3**	**12.8 ± 0.8**	6
	Kv6.3	5.6 ± 2.3	10.1 ± 0.6	7	**13.8 ± 2.0**	**10.9 ± 1.4**	5
	Kv6.4	1.5 ± 1.4	20.0 ± 1.0	9	−0.3 ± 1.9	21.3 ± 2.1	9
	Kv6.4_S6c_Kv9.3	−2.6 ± 1.0	13.2 ± 0.3	8	−0.5 ± 1.2	15.5 ± 0.7	8
	Kv9.3	−5.7 ± 1.7	10.3 ± 0.2	5	−7.0 ± 0.5	8.9 ± 0.6	5
	Kv9.3_S6c_Kv6.4	6.7 ± 1.2	11.0 ± 0.9	6	4.8 ± 1.3	10.2 ± 0.3	6
Inactivation	Kv2.1	−19.4 ± 2.3	6.3 ± 0.5	6	−11.4 ± 1.0	5.6 ± 0.6	6
	Kv6.3	**-**20.0 ± 3.8	6.9 ± 0.4	5	**-4.8 ± 1.5**	**8.0 ± 0.6**	5
	Kv6.4	**−59.0 ± 1.2**	8.4 ± 0.4	7	**−52.0 ± 2.6**	7.7 ± 0.1	7
	Kv6.4_S6c_Kv9.3	−58.5 ± 1.4	7.7 ± 0.2	5	−56.8 ± 1.2	7.9 ± 0.8	5
	Kv9.3	**−48.7 ± 1.4**	7.0 ± 0.5	5	**−45.4 ± 1.2**	7.8 ± 0.7	5
	Kv9.3_S6c_Kv6.4	**−31.3 ± 0.7**	7.3 ± 0.3	5	**−28.7 ± 2.0**	8.4 ± 0.8	5

V_1/2_, midpoint of activation and inactivation; k, slope factor; n, number of cells. Values in bold are significantly different (p < 0.05) compared to control values

While not significantly altering the voltage dependence of activation, 4−AP shifted the voltage dependence of inactivation of Kv2.1/Kv6.4 channels slightly towards more depolarized potentials (p = 0.031, Fig 3D and Table 1). To exclude that this was due to a possible depolarizing drift in our recordings, the V_1/2_ of inactivation was performed over the course of 25 min. (S4A Fig). The V_1/2_ of inactivation displayed a small, non significant drift towards hyperpolarized potentials when comparing the initial recording (t = 5 min.) with the final recording (t = 25 min.) (p = 0.343). However, 4-AP (recorded after such time course analysis) still significantly shifted the inactivation process towards depolarizing potentials (p = 0.019) (S4B and S4C Fig).

Kv2.1/Kv6.4 channels inactivate before the BC gate opens as reflected by the 60 mV difference between the midpoint of the voltage dependence of inactivation and activation. In addition, Kv2.1/Kv6.4 channels possess, as illustrated in panel D of Fig 3 (black circles), a strong U−shaped inactivation profile, indicative of preferential inactivation from closed and intermediate open states while displaying less efficient inactivation from the open state, a process also known as closed-state inactivation [31–34]. Fig 3D reveals that 4−AP not only reduced the amount of Kv2.1/Kv6.4 channel inactivation during the 5 second prepulse but also the degree of U−type inactivation. The reduction in channel inactivation was most pronounced at potentials below the threshold for BC gate opening, i.e. more negative than −40 mV. Under control conditions 12 ± 2, 23 ± 3, 46 ± 5 and 73 ± 3% (n = 8) of Kv2.1/Kv6.4 channels are inactivated at −80, −70, −60 and −50 mV, respectively; 4−AP significantly decreased the amount of inactivation at these potentials to roughly 1 (p = 0.033), 12 ± 3 (p = 0.044), 31 ± 6 (p = 0.041) and 55 ± 7% (p = 0.036), respectively (n = 7) (Fig 3D). This suggested that 4−AP prevented (or reduced) the transition from a closed state to a closed−inactivated state in Kv2.1/Kv6.4 channels. The concentration−dependence of the 4−AP induced current increase saturated at concentrations above 10 mM, EC_50_ = 2.9 ± 0.1 mM (n = 5), but for consistency, all of the following experiments were performed with the 18 mM 4−AP screening concentration (Fig 3E).

### Effect of 4−AP on the kinetics of closed−state inactivation

**Fig 4:**
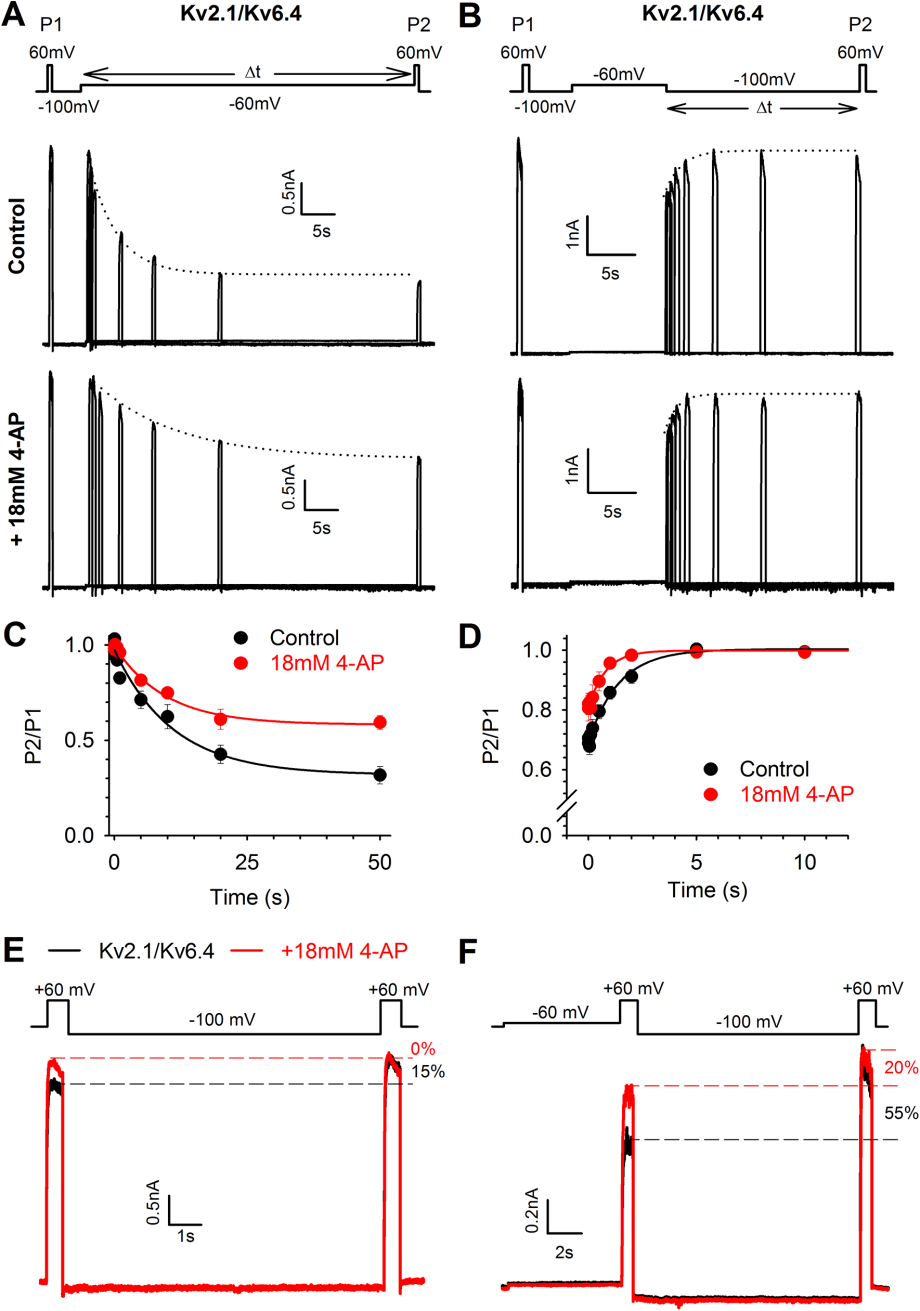
4−AP affects closed−state inactivation in Kv2.1/Kv6.4 heterotetramers. (A−B) Representative current recordings in control conditions (upper) and after application of 18 mM 4−AP (bottom) elicited by the pulse protocol shown in the top to investigate the time course of induction (A) and recovery (B) from closed−state inactivation. The dotted lines represent the exponential fit to determine the closed−state inactivation initiation (A) and recovery (B) rate. (C−D) Induction at −60 mV (C) and recovery at −100 mV (D) of closed−state inactivation obtained by plotting the normalized P2/P1 amplitude in function of time. Solid lines represent the exponential fit. Note that 18 mM 4−AP decreased the amount of inactivation and slowed the initiation rate of closed−state inactivation (C) while accelerating the recovery from closed−state inactivation (D). (E) The twin pulse protocol (top) reveals that under control conditions (black trace) a fraction of channels can be recovered from their closed−inactivated state by a 10s pulse to −100mV, seen as an increase in peak amplitude from P1 to P2. With 4−AP (red trace), the amplitude at both test pulses were the same as P2 in control, suggesting that 4−AP interferes with the transition from the closed to the closed−inactivated state. The holding potential was kept at −80 mV between different current traces. (F) Inducing more closed−state inactivation by a 5s prepulse to −60 mV increased the difference in peak amplitude between P1 and P2 pulse by approximately 50% in control. 4−AP reduced the current less after the prepulse to −60 mV compared to control conditions and reduced the difference in peak amplitude of the test pulse to approximately 15%, suggesting that 4−AP interfered with the closed to closed−inactivated state transition.

4−AP could interfere with the closed to closed−inactivated state transition in Kv2.1/Kv6.4 channels by altering the induction of and/or recovery from closed−state inactivation. To investigate whether 4−AP affects the rate of development of closed−state inactivation, we used a protocol as illustrated in Fig 4A; the initial current amplitude was recorded at a test pulse to +60 mV (P1) followed by a 5s recovering pulse to −100 mV to eliminate any inactivation that had developed, a pulse of variable duration to −60 mV to develop closed−state inactivation and a second test pulse to +60 mV (P2) to obtain the current amplitude after the initiation of closed−state inactivation. The fraction P2/P1 represents the degree of inactivation and was plotted against the time spent at −60 mV (Fig 4C, normalized plot). The holding potential was kept at −100 mV to avoid development of closed−state inactivation between the different current traces. Under control conditions (upper panel Fig 4A, black circles Fig 4C) approximately 70% of the Kv2.1/Kv6.4 channels were inactivated after 50s at −60 mV and closed−state inactivation developed with a time constant of 5.4 ± 0.2 s (n = 6). In the presence of 4−AP (lower panel Fig 4A, red circles Fig 4C), the total amount of inactivation was reduced to approximately 40% and the time constant was increased to 9.3 ± 0.9 s (n = 6, p = 0.032). To investigate the rate of recovery from closed−state inactivation, a similar protocol was used (Fig 4B); the initial +60 mV (P1) and recovery −100 mV pulse remained the same but were now followed by a 10 s pulse to −60 mV to induce closed−state inactivation, followed by a pulse of variable duration to −100 mV to allow the channels to recover from closed−state inactivation and a second test pulse to +60 mV (P2) to obtain the current amplitude after the recovery from closed−state inactivation. The P2/P1 ratio now represents the fraction of Kv2.1/Kv6.4 channels that had recovered from a closed−inactivated state (Fig 4D). In the presence of 4−AP, Kv2.1/Kv6.4 channels recovered faster from closed−state inactivation (τ = 0.5 ± 0.1 s, n = 6) compared to the Kv2.1/Kv6.4 recovery under control conditions (τ = 1.2 ± 0.2s, n = 6, p = 0.009). These results suggest that 4−AP prevented (or reduced) the transition from the closed to closed−inactivated state of Kv2.1/Kv6.4 channels by slowing the induction of closed−state inactivation and increasing the rate of recovery from closed−state inactivation.

To confirm that 4−AP interferes with the transition from the closed state to the closed−inactivated state in Kv2.1/Kv6.4 channels, a twin−pulse protocol was used: two depolarizing pulses to +60 mV were separated by a 10 s interval at −100 mV allowing the Kv2.1/Kv6.4 channels to recover from closed−state inactivation (τ for recovery from closed−state inactivation was 1.2 ± 0.2 s, Fig 4D). Under control conditions, the current amplitude obtained during the second depolarizing pulse was 16.1 ± 0.9% (n = 7) higher than the current amplitude obtained at the first depolarizing pulse (Fig 4E, black trace). However, in the presence of 4−AP no significant differences in current amplitude were observed between the first and second depolarizing pulse and the difference in current amplitude (%) between the control and 4-AP conditions was found to be statistically significant (p < 0.001, Fig 4E, red trace). Furthermore, the peak amplitude of both pulses corresponded to the current amplitude of the second pulse obtained under control condition. These results indicated that a substantial fraction of the Kv2.1/Kv6.4 channels (~15%) reached the closed−inactivated state at a holding potential of −80 mV and that 4−AP prevented (or reduced) this closed state to closed−inactivated state transition due to a faster recovery from and slower initiation of closed−state inactivation (Fig 4C and 4D). To confirm our hypothesis further we promoted closed−state inactivation in Kv2.1/Kv6.4 channels by applying our two−pulse protocol after a 5 s pre−pulse to −60 mV (Fig 4F). This −60 mV pre−pulse induced more closed−state inactivation under control conditions: the current amplitude of the second depolarizing pulse was 56 ± 6% (n = 6) higher than that of the first depolarizing pulse. In presence of 4−AP the difference between the second and first depolarizing test pulse was reduced to only 18 ± 4% (n = 5), as expected if 4−AP reduced the closed−state to closed−inactivated state transition through an increased recovery rate (at -100 mV) from and slower initiation rate (at -60 mV) to the closed−inactivated state (Fig 4F). The difference in current amplitudes (%) between control and 4−AP conditions proved to be statistically significant (p = 0.002).

**Fig 5:**
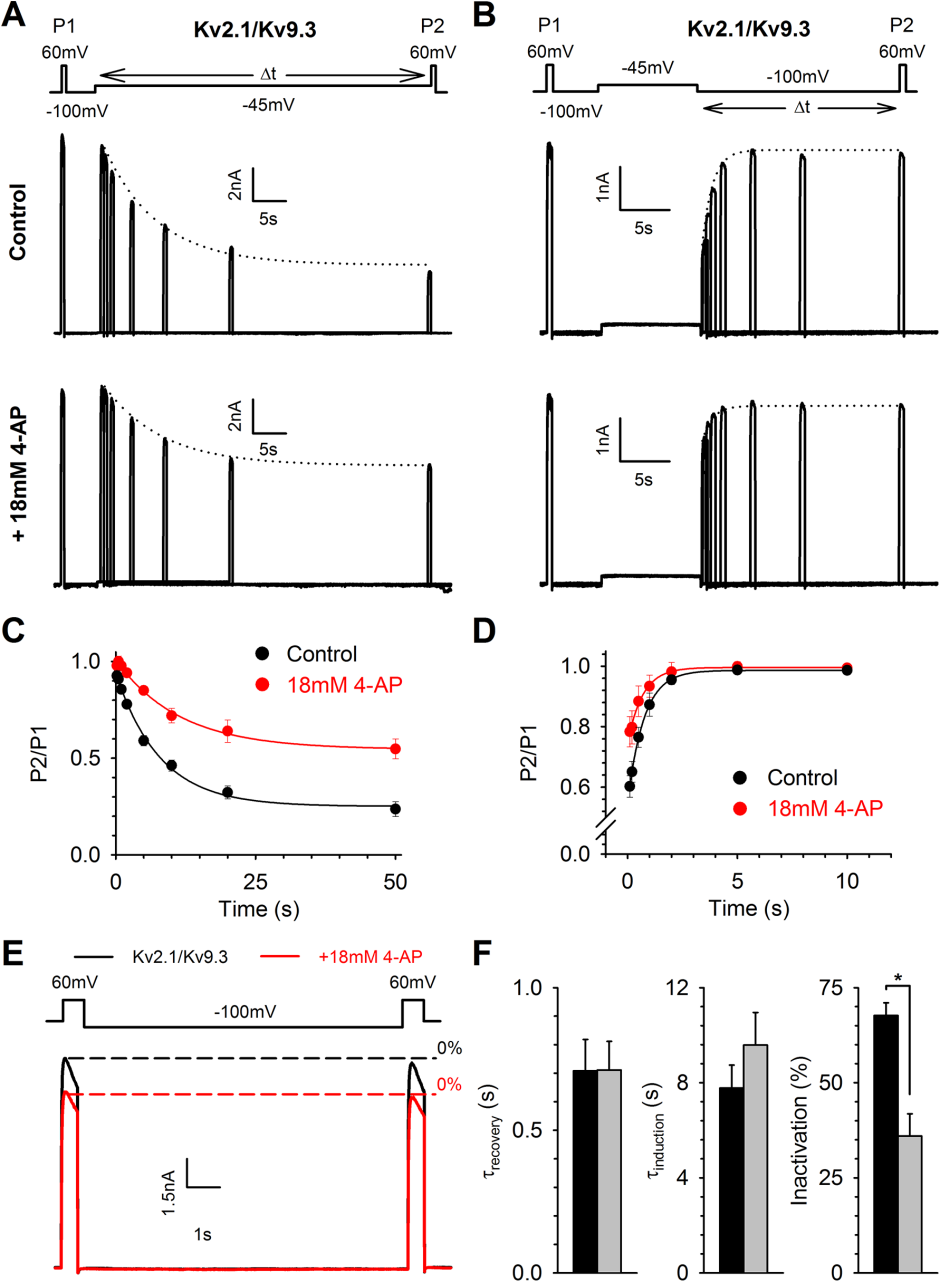
4−AP does not affect closed−state inactivation in Kv2.1/Kv9.3 heterotetramers. (A−B) Representative current recordings under control (upper) and 4−AP (bottom) conditions elicited by the pulse protocols shown on top. The pulse protocols were used to investigate the induction (A) and recovery (B) of closed−state inactivation. The dotted lines represent the exponential fit to determine these rate constants. (C−D) The normalized plot of induction at −45 mV (C) and recovery at −100 mV (D) of closed−state inactivation was obtained as described in Fig 4. Solid lines represent the exponential fit. 4−AP decreased the total amount of inactivation developing at the V_1/2_ of inactivation but did not significantly alter the induction and recovery rate of closed−state inactivation. (E) The twin pulse protocol (top), as described in Fig 4, revealed that under control conditions (black trace) no substantial fraction of Kv2.1/Kv9.3 channels is in a closed−inactivated state as the peak amplitude between P1 and P2 is equal. 4−AP inhibited both P1 and P2 to the same extent. Thus, in contrast to Kv2.1/Kv6.4 channels, no closed−inactivated Kv2.1/Kv9.3 channels can be recovered by 4−AP or the −100 mV recovering pulse. (F) Bar chart of the kinetics of closed−state inactivation before and after application of 4−AP: recovery rate (left), induction rate (middle) and degree of inactivation after a 10s pulse to **−**45 mV (right). * represent statistical significance. Black and grey bars represent control and 18 mM 4−AP, respectively.

Interestingly, some other Kv2.1/KvS and homotetrameric Kv2.1 channels display closed−state inactivation but were inhibited by 4−AP. If the 4-AP induced potentiation and kinetic changes in Kv2.1/Kv6.4 channels reflect interference with closed−state inactivation, then 4−AP should not alter the kinetics of closed−state inactivation of channels that possess a strong preference for closed−state inactivation but are inhibited by 4−AP. Therefore, we investigated the effect of 4−AP on the kinetics of closed−state inactivation (induction and recovery) of heterotetrameric Kv2.1/Kv9.3 (Fig 5) and homotetrameric Kv2.1 (S5 Fig). The same protocols for induction of (Fig 5A, S5A Fig) and recovery from (Fig 5B, S5B Fig) closed−state inactivation were used but the voltage of the step to induce inactivation was changed to match the V_1/2_ of inactivation of the respective channel (i.e. −45 mV for Kv2.1/Kv9.3 and −20 mV for Kv2.1). Interestingly, 4−AP had no effect on the kinetics of the closed−state inactivation of Kv2.1/Kv9.3 (Fig 5C, 5D and 5F) and Kv2.1 (S5C, S5D and S5F Fig) channels although it did reduce the degree of inactivation (Figs 5C, 5D and 5F, S5C, S5D and S5F). Thus, 4−AP reduced the degree of inactivation developing at the V_1/2_ of inactivation–when superimposed on the inhibition − but was not capable of altering the kinetics of closed−state inactivation in Kv2.1/Kv9.3 and Kv2.1 channels, suggesting that 4−AP interferes with the closed−state inactivation process of Kv2.1/Kv6.4 channels.

### The S6c sequence affects the Kv6.4 specific response to 4−AP

**Fig 6:**
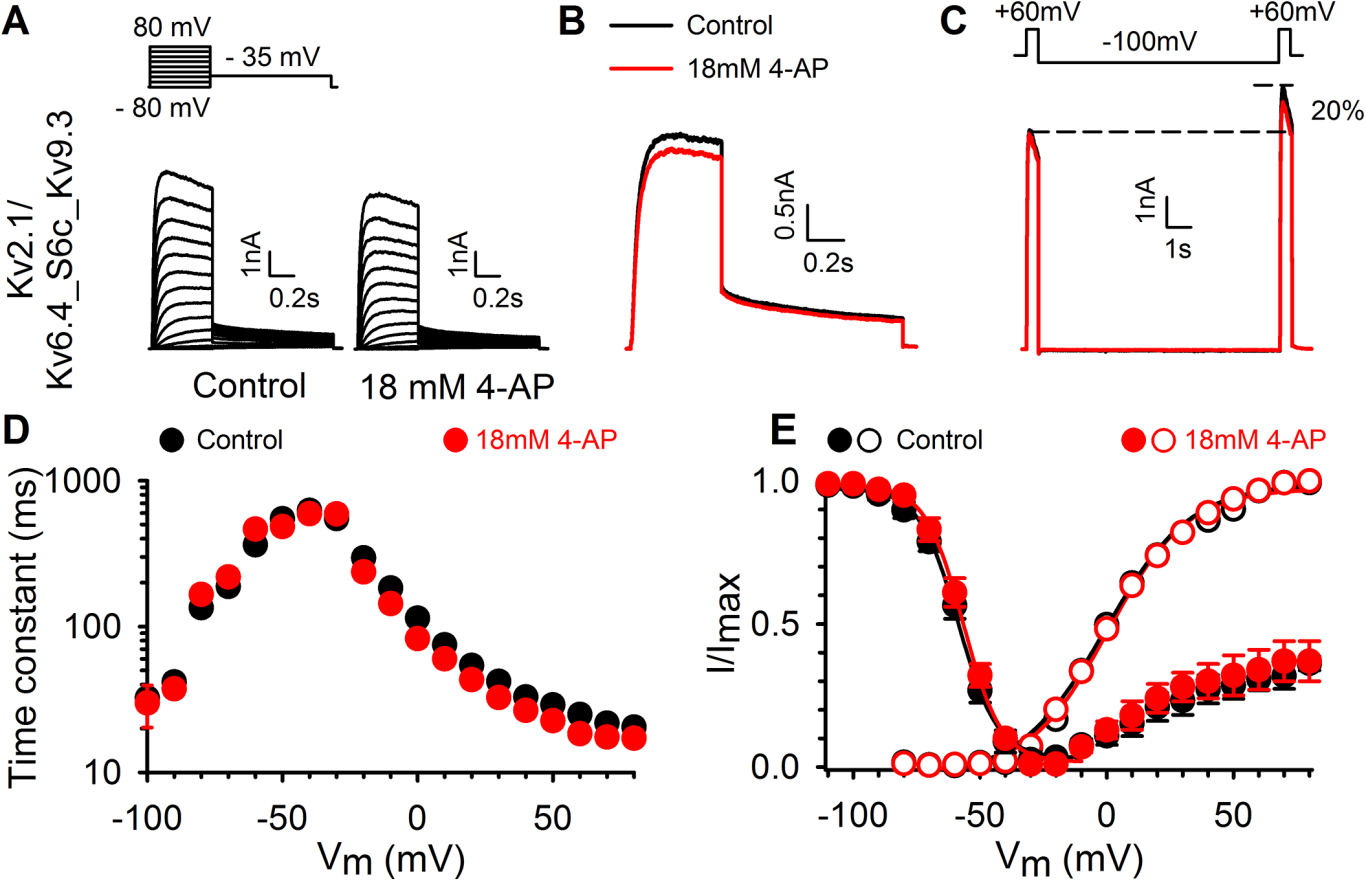
Involvement of the S6c domain in the KvS specific response to 4−AP. (A) Typical current traces for the Kv2.1/Kv6.4_S6c_Kv9.3 chimera without (left) and with 4−AP (right) revealing inhibition by 4−AP, comparable to its effect on Kv2.1/Kv9.3_WT currents. The pulse protocol is shown above the current traces. (B) 4−AP (red trace) causes a small inhibiting effect of Kv2.1/Kv6.4_S6c_Kv9.3 currents which is the opposite of the 4−AP effect on the Kv2.1/Kv6.4_WT currents. Control trace shown in black. (C) The twin pulse protocol from Fig 4E was repeated on the Kv6.4_S6c_Kv9.3 chimera to illustrate that: 1) closed−state inactivation is still present at −80mV, 2) inactivated channels can be recovered in a 10s pulse to −100 mV and 3) 4−AP is unable to interfere with the closed−state inactivation of the Kv6.4_S6c_Kv9.3 chimera. (D) Activation and deactivation kinetics of the Kv2.1/Kv6.4_S6c_Kv9.3 heterotetramers (black circles). Time constants of the chimera were obtained with a single exponential function which resulted in a single fast component that remained unaltered under the influence of 4−AP (red circles). This chimera lacked the pronounced slow component present in Kv2.1/Kv6.4_WT heterotetramers (Fig 3C). (E) Voltage−dependence of activation (open symbols) and inactivation (closed symbols) of the Kv2.1/Kv6.4_S6c_Kv9.3 chimera obtained like for the WT Kv2.1/Kv6.4 heterotetramers in Fig 3. Both were nearly the same as for WT Kv2.1/Kv6.4 currents and remained unaffected by 4−AP application (red symbols).

Exchanging the S6c sequence between Kv2.1 and Kv3.1 has been shown to transfer their 4−AP sensitivity [27]. Similar to Kv6.4, Kv9.3 induces a negative shift in the voltage−dependence of inactivation but Kv2.1/Kv9.3 currents are blocked by 4−AP (Fig 2). Therefore, the potentiation of Kv2.1/Kv6.4 currents by 4−AP appears to be a specific feature of the Kv6.4 subunit. To test whether the S6c sequence of the Kv6.4 subunit confers 4−AP induced potentiation, we replaced the S6c of Kv6.4 by that of Kv9.3 (chimera Kv6.4_S6c_Kv9.3). This Kv6.4_S6c_Kv9.3 chimera yielded functional heterotetrameric channels upon co−expression with Kv2.1, and displayed slightly altered activation kinetics compared to Kv2.1 heterotetrameric channels composed of either Kv6.4 or Kv9.3 (Fig 6A and 6D). The Kv2.1/Kv6.4_S6c_Kv9.3 channels displayed a voltage dependence of activation similar to both Kv2.1 homomeric channels and WT Kv2.1/Kv6.4 or Kv2.1/Kv9.3 heterotetramers (Fig 6E and Table 1). Additionally, the characteristic inactivation from the closed state was preserved in the chimera and closely resembled that of WT Kv2.1/Kv6.4 heterotetramers (Fig 6C and 6E). Application of 18 mM 4−AP caused an inhibition of Kv2.1/Kv6.4_S6c_Kv9.3 currents of 12 ± 3% (n = 7), which is comparable to the 12 ± 1% inhibition of WT Kv2.1/Kv9.3 heterotetramers (p = 0.295) and in marked contrast to the activator effect in WT Kv2.1/Kv6.4 channels (Fig 6A and 6B). 4−AP did not affect the voltage dependence of channel inactivation (p = 0.582). These results showed that exchanging the S6c region from Kv6.4 to Kv9.3 transferred the 4−AP effect of Kv9.3 to the Kv6.4 subunit resulting in current inhibition.

The opposite effect might be expected when introducing the S6c sequence of Kv6.4 in Kv9.3 (chimera Kv9.3_S6c_Kv6.4). This Kv9.3_S6c_Kv6.4 chimera also yielded functional heterotetrameric channels upon co−expression with Kv2.1 (S6A Fig) but displayed a significantly altered voltage dependence of activation (p < 0.001) and inactivation (p = 0.002), compared to wilt−type Kv2.1/Kv9.3 heterotetramers. Furthermore, the gating of Kv2.1/Kv9.3_S6c_Kv6.4 channels significantly differed from the voltage−dependence of activation (p = 0.002) and inactivation (p = 0.001) of homotetrameric Kv2.1 channels. The Kv2.1/Kv9.3_S6c_Kv6.4 channels displayed a voltage dependence of activation and inactivation that was shifted by approximately 10 and 20 mV towards depolarized potentials, respectively (S6E Fig and Table 1). Due to the shifted voltage dependency of inactivation and the reduction of the U−shaped inactivation profile, it appears that the characteristic closed−state inactivation was lost in Kv2.1/Kv9.3_S6c_Kv6.4 heterotetramers. Therefore, 4−AP might have no potentiating effect on Kv2.1/Kv9.3_S6c_Kv6.4 channels as 4−AP affects the closed to closed−inactivated state transition. Indeed, no difference in current amplitude can be observed between the 1^st^ and 2^nd^ depolarizing pulse to + 60 mV under control conditions (S6C Fig). Furthermore, 4−AP inhibited both pulses of the twin pulse protocol but the ratio between the 1^st^ and 2^nd^ depolarizing pulse remained the same. The combined results of the Kv6.4_S6c_Kv9.3 and Kv9.3_S6c_Kv6.4 chimera’s illustrate that potentiation of Kv6.4 currents may involve the presence of the Kv6.4’s S6c sequence and a strong closed−state inactivation process.

## Discussion

### 4−AP potentiates currents mediated by Kv2.1/Kv6.4 heteromeric channels

The S6c domain has been shown to form a binding site for channel inhibitors such as 4−AP, flecainide, propafenone and quinidine [9–12,35]. The S6c of most Kv channels contains a conserved PXP motif that, besides being important for channel gating [6,36], has been shown to modulate the channel´s sensitivity to 4−AP and internal pore blockers (e.g. quinidine). Substituting the second proline of the PXP motif by an alanine drastically decreased the affinity for 4−AP in *Shaker* [25]. Interestingly, all the members of the Kv7 (KCNQ) subfamily lack a full PXP motif (Fig 1) and neither of them is sensitive to 4−AP [37]. The fact that the KvS subunits also miss the 2^nd^ proline of the PXP motif (Fig 1) raised the question whether heteromeric Kv2.1/KvS channels display a different pharmacological profile for 4−AP compared to homomeric Kv2.1 channels.

To address this question we tested 4−AP on the different KvS subfamilies using Kv5.1, Kv6.4, Kv8.1 and Kv9.3 as representative members. Our data show that Kv5.1, Kv8.1 and Kv9.3 heteromeric Kv2.1 channels displayed a reduced affinity for 4−AP compared to Kv2.1 homotetramers (Fig 2C), which is in agreement with previous findings on Kv9.3 channels [16]. However, the most intriguing change in 4−AP sensitivity was induced by the Kv6.4 subunit: in this case 4−AP potentiated, rather than inhibited, Kv6.4 currents. Interestingly, in mouse bladder smooth muscle cells and gastrointestinal smooth muscle cells it has been demonstrated that 4-AP was capable of potentiating native Kv currents [38,39]. In addition, the expression of several KvS subunits was confirmed in mouse bladder smooth muscle cells [38,40]. Importantly, Thorneloe and Nelson documented both the presence of Kv6.4 (in their study named Kv6.3 following the old nomenclature) in isolated bladder myocytes, and potentiation of the delayed rectifier Kv current by 10 mM 4-AP in these cells, thus providing physiological relevance for our heterologously obtained data [38].

The potentiation was a unique characteristic of 4−AP since the internal pore blockers quinidine and flecainide still blocked Kv2.1/Kv6.4 channels, although with a slightly higher affinity compared to homotetrameric Kv2.1 channels (S2C Fig). A similar increase in quinidine affinity was also observed in the *Shaker* mutant with a PXA sequence [25]. The potential to convert the 4−AP effect from inhibition to current potentiation appears not to be conserved within all members of the Kv6 family since a previous study reported that Kv2.1/Kv6.2 heterotetramers display a higher degree of current inhibition by 18 mM 4−AP compared to Kv2.1 homotetramers [41]. We observed that Kv2.1/Kv6.3 heterotetramers are also inhibited by 4-AP (S1 Fig) strengthening the fact that the 4-AP induced current potentiation is not conserved in the Kv6 subfamily. Note that Kv2.1/Kv6.3 channels do not display a major hyperpolarizing shift of the inactivation curve, as previously reported by Ottschytsch et al. (then designated as Kv10.1)[15]. Thus, each Kv2.1/KvS heterotetramer may harbor a unique affinity for 4−AP different from Kv2.1 homotetramers.

### 4−AP interferes with closed−state inactivation transition

In heterotetrameric Kv2.1/Kv6.4 channels, the voltage dependence of inactivation is shifted by 40 mV towards more negative potentials without substantially affecting the voltage dependence of activation. Consequently, the Kv2.1/Kv6.4 channels start to inactivate at potentials that are below the apparent threshold for channel activation. Typically, ion channels with preferential closed−state inactivation possess a U− (or J) shaped (steady state) inactivation profile, i.e. at stronger depolarizations the degree of inactivation diminishes, a characteristic strongly present in Kv2.1/Kv6.4 channels [32,42–44]. At −80 mV, approximately 12% of the Kv2.1/Kv6.4 channels populate the closed−inactivated state under control conditions, as evidenced using a twin pulse protocol in Fig 4E, and 4−AP restored this difference in current amplitude suggesting a possible interference with closed−state inactivation. An extra 5 s prepulse to −60 mV in the twin pulse protocol increased the total amount of closed−state inactivation and allowed 4−AP to recover more Kv2.1/Kv6.4 channels from their inactivated state, strengthening this hypothesis (Fig 4F). If Kv2.1/Kv6.4 channels exhibit a small, non zero open probability (P_o_) at −60 mV, it cannot be excluded that, in combination with the duration of the conditioning pulse, 4−AP (partly) acts on open−state inactivation, which has been previously reported for the *Shaker* channel [45]. However, the observations that 4−AP reduced and slowed induction of inactivation, enhanced the rate of recovery and diminished the degree of U−shaped inactivation all points towards a loss of closed−state inactivation and more inactivation at positive potentials (e.g. +80 mV). The latter observation can be explained by a strengthening of open−state inactivation, as suggested by the macroscopic current kinetics in Fig 3A, which is in contrast to previous findings that 4−AP can reduce open−state inactivation [45]. The apparent increase in open−state inactivation could be a consequence of open−channel block however this is rather unlikely due to the potentiation of the peak current amplitude. In summary, 4−AP most likely interferes with closed−state inactivation (Figs 3D and 4A–4D) and the ‘loss’ of closed−state inactivation allows the Kv2.1/Kv6.4 channels to inactivate from their open−state. Hence, the current potentiation observed for Kv2.1/Kv6.4 channels is the result of a reduction of the amount of Kv2.1/Kv6.4 channels present in a closed−inactivated state at the holding potential of −80 mV.

The exact underlying mechanism of closed−state inactivation has not been resolved yet, but several studies indicate that it involves a dynamic coupling between S6c and the S4−S5 linker, which forms the electromechanical coupling that translates VSD movement into BC gate opening [43,46]. If an uncoupling occurs at this S6c/S4−S5linker interface, BC gate (and channel) opening is prevented and the channel enters a nonconductive, inactivated state. Due to the subunit−cooperative nature of BC gate opening in *Shaker*−related Kv channels, disrupting the electromechanical coupling of one subunit (e.g. Kv6.4) would be sufficient to enter this nonconductive state. A similar mechanism of uncoupling has been proposed for hyperpolarized cyclic nucleotide−gated (HCN) channels where the uncoupling between the VSD and the channel gate (at hyperpolarized potentials) results in desensitization to voltage, observed as gating charge immobilization [47,48]. Interestingly, such a desensitization to voltage, observed as gating charge immobilization, also occurs in Kv4.2 channels that exhibit prominent closed−state inactivation [49].

If closed−state inactivation involves a collapse of the selectivity filter, as in C−type inactivation, then the process is most likely triggered by rearrangements in S6c (in the vicinity of the BC gate). Indeed, in C−type inactivation the S6c reorientations associated with BC gate opening are transmitted upwards along the S6 helix up to the level of the selectivity filter [50–52]. The final collapse of the selectivity filter is probably a process whereby the destabilizing input of 1 subunit is sufficient to trigger inactivation [53,54]. If closed−state inactivation has a similar mechanism, the rearrangements in S6c should occur, and be transmitted upwards, before channel opening. For the Kv2.1/Kv6.4 heteromeric channels we showed that the gating charge movement displays a component at hyperpolarized potentials with a voltage dependence that matches the voltage−dependence of inactivation [55]. This suggests that at −80 mV already a substantial amount of the gating charge moves (originating from the S4 movement of either the Kv6.4 or Kv2.1 subunits) which could potentially be translated in conformational changes of S6c associated with inactivation gating. It is currently not known whether the S6c of Kv6.4 actually moves, but if it does and if this movement occurs at more hyperpolarized potentials, channel inactivation could be triggered. In this model our data would strengthen the hypothesis that one subunit is sufficient to initiate the collapse of the selectivity−filter [50,51].

Here we propose that 4−AP modulates closed−state inactivation in the heteromeric Kv2.1/Kv6.4 channels either by stabilizing the coupling at the S6c/S4−S5linker interface or by preventing S6c reorientations associated with inactivation gating. In tuberomammillary neurons, it has been demonstrated that 4−AP enhanced the Kv4 A−type current (I_A_) at subtreshold potentials [56]. It was suggested that 4−AP−bound channels were unable to inactivate resulting in a potentiation at subthreshold potentials. This observation fits with our finding that 4−AP prevents closed−sate inactivation, a process that is also present in Kv4.2 channels [31].

### Molecular determinants of the 4−AP binding site

Location of the 4−AP binding site remains uncertain but both the S4−S5 linker and the cytoplasmic half of S5 and S6 have been shown to be important for 4−AP sensitivity in Kv channels [10,27,30,57]. Molecular docking studies of aminopyridines confirmed this and placed the 4−AP binding pocket at the S4−S5 linker and S6c interface [58]. However, the location of 4−AP binding site does not allow us to rule out whether closed−state inactivation arises from uncoupling the S6c/S4−S5linker interface or conformational changes that lead to selectivity filter collapse. In both cases, 4−AP unbinding is required in order for closed−state inactivation to occur.

Kv2.1/Kv9.3 heteromers display a large hyperpolarizing shift in the voltage−dependence of inactivation (similar to Kv2.1/Kv6.4 heteromers), but they are inhibited, rather than potentiated by 4−AP. This suggests that the hyperpolarized position of the inactivation curve is not the only determinant for the potentiating effect of 4−AP. Kirsch and Drewe were able to transfer the high affinity of Kv3.1 for 4−AP onto Kv2.1 by exchanging both the S5 and S6 domains [27]. Given the sequence differences in S6c between Kv6.4 and Kv9.3 we exchanged this region and found that substituting S6c in Kv6.4 by the Kv9.3 sequence (Kv2.1/Kv6.4_S6c_Kv9.3) transferred Kv9.3´s 4−AP effect onto Kv6.4 subunits, i.e. the Kv2.1/Kv6.4_S6c_Kv9.3 channels were inhibited by 4−AP instead of potentiated (Fig 6). However, the opposite substitution, i.e. replacing the S6c region of Kv9.3 by the Kv6.4 sequence, did not transfer the 4−AP potentiation effect onto the Kv9.3 currents. Interestingly, the Kv2.1/Kv9.3_S6c_Kv6.4 channels displayed altered biophysical properties, including a depolarizing shift of approximately 20mV in the voltage dependence of inactivation and a reduced degree of U−shaped inactivation. This suggests that the 4−AP effect on Kv2.1/KvS heterotetramers is dependent on both the S6c sequence and the presence or absence of the prominent closed−state inactivation process. It cannot be excluded that other channel regions are involved in creating the 4−AP binding site, especially the S4−S5 linker and the cytoplasmic half of S5 due to their contribution to dynamic coupling in the known mechanism of closed−state inactivation.

### General conclusion

Our present study reports that KvS subunits have the ability to alter the pharmacological profile of Kv2.1 channels upon heterotetramerization. Thus, heterotetrameric Kv2/KvS channels not only display unique biophysical properties but also unique pharmacological effects as is the case for 4−AP on Kv2.1/Kv6.4 channels where it interferes with the closed−state inactivation process. It has been demonstrated that heterotetrameric Kv2.1/KvS channels regulate several (patho)physiological processes, e.g. mutations of Kv8.2 contribute to epilepsy susceptibility, as they contribute to several native delayed rectifier currents [19,20,59,60]. Because of their structural differences and unique biophysical properties, different KvS subunits can not only alter the Kv2 pharmacology, but it is also likely that KvS subunits could be pharmacologically specifically targeted, without influencing Kv2 homomers, allowing interventions in physiological processes regulated by unique Kv2/KvS heteromers.

## Supporting Information

S1 Fig 4-AP inhibits heterotetrameric Kv2.1/Kv6.3 channels. **(**A) Representative current recordings of Kv2.1/Kv6.3 before (left) and after application of 4-AP (right). 4-AP inhibited Kv2.1/Kv6.3 currents and modified the activation kinetics. Voltage protocol is shown on top. (B) Voltage dependence of activation with (red) and without 4-AP (black). 4-AP slightly shifted the activation curve towards depolarized potentials. (C) Concentration-dependence of the 4-AP inhibition. The blocking potency of 4-AP on Kv2.1/Kv6.3 is decreased in comparison to homotetrameric Kv2.1 channels (dotted line). The solid line represent a fit with the Hill equation with an IC_50_ of 15.5 ± 2.9 mM (n = 5). (D) Activation and deactivation kinetics of the Kv2.1/Kv6.3 heterotetramers. Time constants were fitted with a double exponential function and yielded a fast (circles) and slow (triangles) component. 4-AP (red) slowed both the fast and slow component of activation while the deactivation components remained unaffected. (E) The voltage dependence of inactivation before (black) and after 4-AP application (red). Kv6.3 does not affect the inactivation properties significantly as the inactivation curve overlaps completely with that of homotetrameric Kv2.1 channels (dotted line). 4-AP shifted the inactivation curve towards depolarized potentials and the extent of the shift was comparable to the shift of the activation curve.
(TIF)

S2 Fig Kv2.1/Kv6.4 channels remain sensitive to the channel blockers quinidine and flecainide. (A) Representative current recording before (black trace) and after 3 μM quinidine (red traces) of Kv2.1 (left) and Kv2.1/Kv6.4 channels (right). (B) Concentration dependence for inhibition by quinidine of Kv2.1 (filled circles) and Kv2.1/Kv6.4 (open circles) channels. Concentration−effect curves were obtained by plotting the normalized amount of block in function of the applied concentration and fitted with the Hill equation as described in the material and method section. Note the leftward shift of the Kv2.1/Kv6.4 concentration−effect curve compared to that of Kv2.1 homotetramers which corresponds to a twofold difference in IC_50_ value. (C) Effect of flecainide on Kv2.1 (left) and Kv2.1/Kv6.4 (right) channels. (D) Concentration−effect curves for flecainide inhibition of Kv2.1 (filled circles) and Kv2.1/Kv6.4 (open circles) channels obtained like the quinidine concentration−effect curve in panel B. As for quinidine, the heteromeric Kv2.1/Kv6.4 channels are more sensitive to flecainide.
(TIF)

S3 Fig Effects of 4−AP on homotetrameric Kv2.1 channels. **(**A) Typical Kv2.1 current traces without (left) and with (right) 18 mM 4−AP revealing strong current inhibition and changes in activation kinetics. The pulse protocol is shown above the current traces. (B) Voltage dependence of activation for Kv2.1 obtained like for the WT Kv2.1/Kv6.4 heterotetramers in Fig 3. (C) Activation and deactivation kinetics of Kv2.1. Time constants were obtained with a single exponential function as previously described for the Kv2.1/Kv6.4_S6c_Kv9.3 chimera in Fig 6. Kv2.1 kinetics are shown as black circles and as red circles when 4−AP is present.
(TIF)

S4 Fig Time dependent analysis of the inactivation properties of Kv2.1/Kv6.4. (A) Voltage dependence of inactivation, recorded as a function of time. Five inactivation protocols were recorded over a time span of 25 minutes and evaluated for a potential drift of the V_1/2_ of inactivation. A slight hyperpolarizing drift of the inactivation curve can be observed. (B) Comparison of the voltage dependence of inactivation before and after 4−AP application. After recording 5 repetitive inactivation protocols, 4−AP still shifted the inactivation curve towards depolarized potentials. (C) Bar chart showing the mean V_1/2_ of inactivation and S.E.M. at the different time intervals. 4-AP still shifted the V_1/2_ of inactivation significantly (* represent statistical significance) towards depolarized potentials. Colored dots/bars represent inactivation recorded, after obtaining a ‘whole cell’ patch configuration, at: 5 (black), 10 (red), 15 (blue), 20 (green) and 25 (orange) minutes. 4-AP data is shown in grey.
(TIF)

S5 Fig Closed−state inactivation kinetics of Kv2.1 homotetramers are not modulated by 4−AP. (A and B) Representative current recordings in control conditions (upper) and after application of 18 mM 4−AP (bottom) elicited by the pulse protocol shown on top to investigate the time course of induction (A) and recovery (B) from closed−state inactivation. The dotted lines represent the exponential fit to determine the closed−state inactivation induction (A) and recovery (B) rate. (C and D) Induction at −20 mV (C) and recovery at −100 mV (D) of closed−state inactivation where determined as described in Fig 4. Solid lines represent the exponential fit. 4-AP did not alter the induction or recovery rate of closed−state inactivation. However, 18 mM 4−AP significantly reduced the degree of inactivation that developed during the protocols. (E) Twin pulse protocol (top), as used in Fig 4, revealed that under control (black trace) and 4−AP (red trace) conditions no fraction of channels can be recovered from their closed−inactivated state by a 10 s pulse to −100 mV. Thus, at the holding potential of −80 mV, no substantial amount of closed−state inactivation develops for homotetrameric Kv2.1 channels. (F) Bar chart of the kinetics of closed−state inactivation before and after application of 4−AP: induction rate (left), recovery rate (middle) and degree of inactivation after a 20 s pulse to −20 mV (right). * represent statistical significance). Black and grey bars represent control and 18 mM 4−AP, respectively.
(TIF)

S6 Fig Kv2.1/Kv9.3_S6c_Kv6.4 heterotetramers are inhibited by 4−AP. (A) Typical current traces for the Kv2.1/Kv9.3_S6c_Kv6.4 chimera without (left) and with 4−AP (right). The pulse protocol is shown on top. (B) 4−AP (red trace) slightly inhibited Kv2.1/Kv9.3_S6c_Kv6.4 currents (17 ± 3%, n = 6) comparable to the 12 ± 1% inhibition of WT Kv2.1/Kv9.3 currents seen in Fig 2. Control trace shown in black. (C) The twin pulse protocol from Fig 4E illustrates that the prominent closed−state inactivation was no longer present in Kv2.1/Kv9.3_S6c_Kv6.4 heterotetramers, seen as equal peak amplitudes at the two test pulses P1 and P2. 4−AP inhibited peak current amplitudes at P1 and P2 to a similar extent. (D) Activation and deactivation kinetics of the Kv2.1/Kv9.3_S6c_Kv6.4 heterotetramers (black circles). Time constants of activation were obtained with a single exponential function. Similar to the Kv2.1/Kv6.4_S6c_Kv9.3 chimera in Fig 6, the Kv2.1/Kv9.3_S6c_Kv6.4 chimera lacked a pronounced slow component present in Kv2/Kv6.4 heterotetramers (Fig 3C). (E) Voltage dependence of activation (open symbols) and inactivation (closed symbols) of the Kv2.1/Kv9.3_S6c_Kv6.4 chimera. Both were shifted towards depolarized potentials compared to the WT Kv2.1/Kv9.3 inactivation (dashed line), resulting in a loss of the pronounced closed−state inactivation process. 4−AP (red symbols) had no significant effect on the gating parameters. For comparison, homomeric Kv2.1 (dotted line) before 4−AP application is included.
(TIF)

## References

[1] Hille B. Ion channels of excitable membranes 3rd ed. Sunderland, Massachusetts: Sinauer; 2001.

[2] Long SB, Campbell EB, MacKinnon R. Crystal structure of a mammalian voltage-dependent Shaker family K^+^ channel. Science 2005;309: 897–903. 16002581 10.1126/science.1116269

[3] Kitaguchi T, Sukhareva M, Swartz KJ. Stabilizing the closed S6 gate in the Shaker Kv channel through modification of a hydrophobic seal. J. Gen. Physiol. 124: 319–332. 15365093 10.1085/jgp.200409098 PMC2233904

[4] Hackos DH, Chang TH, Swartz KJ. Scanning the intracellular S6 activation gate in the Shaker K(+) channel. J. Gen. Physiol. 2002;119: 521–532. 12034760 10.1085/jgp.20028569 PMC2233862

[5] Tieleman DP, Shrivastava IH, Ulmschneider MR, Sansom MS. Proline-induced hinges in transmembrane helices: possible roles in ion channel gating. Proteins 2001;44: 63–72. 11391769 10.1002/prot.1073

[6] Labro AJ, Raes AL, Bellens I, Ottschytsch N, Snyders DJ. Gating of Shaker-type channels requires the flexibility of S6 caused by prolines. J. Biol. Chem. 2003;278: 50724–50731. 13679372 10.1074/jbc.M306097200

[7] Swartz KJ. Structure and anticipatory movements of the S6 gate in Kv channels. J. Gen. Physiol. 2005;126: 413–417. 16260835 10.1085/jgp.200509430 PMC2266603

[8] Baukrowitz T, Yellen G. Two functionally distinct subsites for the binding of internal blockers to the pore of voltage-activated K^+^ channels. Proc. Natl. Acad. Sci. U.S.A. 1996;93: 13357–13361. 8917595 10.1073/pnas.93.23.13357 PMC24097

[9] Yeola SW, Rich TC, Uebele VN, Tamkun MM, Snyders DJ. Molecular analysis of a binding site for quinidine in a human cardiac delayed rectifier K^+^ channel. Role of S6 in antiarrhythmic drug binding. Circ. Res. 1996;78: 1105–1114. 8635242 10.1161/01.res.78.6.1105

[10] Shieh CC, Kirsch GE. Mutational analysis of ion conduction and drug binding sites in the inner mouth of voltage-gated K^+^ channels. Biophys. J. 1994;67: 2316–2325. 7696472 10.1016/S0006-3495(94)80718-0 PMC1225616

[11] Zhang H, Zhu B, Yao JA, Tseng GN. Differential effects of S6 mutations on binding of quinidine and 4-aminopyridine to rat isoform of Kv1.4: common site but different factors in determining blockers' binding affinity. J. Pharmacol. Exp. Ther. 1998;287: 332–343. 9765354

[12] Decher N, Kumar P, Gonzalez T, Pirard B, Sanguinetti MC. Binding site of a novel Kv1.5 blocker: a "foot in the door" against atrial fibrillation. Mol. Pharmacol. 2006;70: 1204–1211. 16835355 10.1124/mol.106.026203

[13] Gutman GA, Chandy KG, Grissmer S, Lazdunski M, McKinnon D, Pardo LA, et al International Union of Pharmacology. LIII. Nomenclature and molecular relationships of voltage-gated potassium channels. Pharmacol. Rev. 2005;57: 473–508. 16382104 10.1124/pr.57.4.10

[14] Bocksteins E, Snyders DJ. Electrically silent Kv subunits: their molecular and functional characteristics. Physiology (Bethesda) 2012;27: 73–84. 22505664 10.1152/physiol.00023.2011

[15] Ottschytsch N, Raes A, Van Hoorick D, Snyders DJ. Obligatory heterotetramerization of three previously uncharacterized Kv channel alpha-subunits identified in the human genome. Proc. Natl. Acad. Sci. U.S.A. 2002;99: 7986–7991. 12060745 10.1073/pnas.122617999 PMC123007

[16] Patel AJ, Lazdunski M, Honore E. Kv2.1/Kv9.3, an ATP-dependent delayed-rectifier K^+^ channel in pulmonary artery myocytes. Ann. N.Y. Acad. Sci. 1999;868: 438–441. 10414317 10.1111/j.1749-6632.1999.tb11309.x

[17] Coppock EA, Martens JR, Tamkun MM. Molecular basis of hypoxia-induced pulmonary vasoconstriction: role of voltage-gated K+ channels. Am. J. Physiol. Lung Cell. Mol. Physiol. 2001;281: L1–12. 11404238 10.1152/ajplung.2001.281.1.L1

[18] Wu H, Cowing JA, Michaelides M, Wilkie SE, Jeffery G, Jenkins SA, et al Mutations in the gene KCNV2 encoding a voltage-gated potassium channel subunit cause "cone dystrophy with supernormal rod electroretinogram" in humans. Am. J. Hum. Genet. 2006;79: 574–579. 16909397 10.1086/507568 PMC1559534

[19] Jorge BS, Campbell CM, Miller AR, Rutter ED, Gurnett CA, Vanoye CG, et al Voltage-gated potassium channel KCNV2 (Kv8.2) contributes to epilepsy susceptibility. Proc. Natl. Acad. Sci. U.S.A. 2011;108: 5443–5448. 10.1073/pnas.1017539108 21402906 PMC3069171

[20] Muller D, Cherukuri P, Henningfeld K, Poh CH, Wittler L, Grote P, et al Dlk1 promotes a fast motor neuron biophysical signature required for peak force execution. Science 2014;343: 1264–1266. 10.1126/science.1246448 24626931

[21] Bocksteins E, Ottschytsch N, Timmermans JP, Labro AJ, Snyders DJ. Functional interactions between residues in the S1, S4, and S5 domains of Kv2.1. Eur. Biophys. J. 2011;40: 783–793. 10.1007/s00249-011-0694-3 21455829

[22] Ottschytsch N, Raes AL, Timmermans JP, Snyders DJ. Domain analysis of Kv6.3, an electrically silent channel. J. Physiol. 2005;568: 737–747. 16096342 10.1113/jphysiol.2005.090142 PMC1464172

[23] Rich TC, Snyders DJ. Evidence for multiple open and inactivated states of the hKv1.5 delayed rectifier. Biophys. J. 1998;75: 183–195. 9649378 10.1016/S0006-3495(98)77505-8 PMC1299690

[24] Snyders DJ, Tamkun MM, Bennett PB. A rapidly activating and slowly inactivating potassium channel cloned from human heart. Functional analysis after stable mammalian cell culture expression. J. Gen. Physiol. 1993;101: 513–543. 8505626 10.1085/jgp.101.4.513 PMC2216772

[25] Martinez-Morales E, Snyders DJ, Labro AJ. Mutations in the S6 gate isolate a late step in the activation pathway and reduce 4-AP sensitivity in Shaker Kv channel. Biophys. J. 2014;106: 134–144. 10.1016/j.bpj.2013.11.025 24411245 PMC3907211

[26] Gutman GA, Chandy KG, Adelman JP, Aiyar J, Bayliss DA, Clapham DE, et al International Union of Pharmacology. XLI. Compendium of voltage-gated ion channels: potassium channels. Pharmacol. Rev. 2003;55: 583–586. 14657415 10.1124/pr.55.4.9

[27] Kirsch GE, Shieh CC, Drewe JA, Vener DF, Brown AM. Segmental exchanges define 4-aminopyridine binding and the inner mouth of K^+^ pores. Neuron 1993;11: 503–512. 8398143 10.1016/0896-6273(93)90154-j

[28] Baranauskas G, Tkatch T, Surmeier DJ. Delayed rectifier currents in rat globus pallidus neurons are attributable to Kv2.1 and Kv3.1/3.2 K^+^ channels. J. Neurosci. 1999;19: 6394–6404. 10414968 10.1523/JNEUROSCI.19-15-06394.1999 PMC6782822

[29] Archer SL, Souil E, Dinh-Xuan AT, Schremmer B, Mercier JC, El Yaagoubi A, et al Molecular identification of the role of voltage-gated K^+^ channels, Kv1.5 and Kv2.1, in hypoxic pulmonary vasoconstriction and control of resting membrane potential in rat pulmonary artery myocytes. J. Clin. Invest. 1998;101: 2319–2330. 9616203 10.1172/JCI333 PMC508821

[30] Kirsch GE, Drewe JA. Gating-dependent mechanism of 4-aminopyridine block in two related potassium channels. J. Gen. Physiol. 1993;102: 797–816. 8301258 10.1085/jgp.102.5.797 PMC2229176

[31] Bahring R, Covarrubias M. Mechanisms of closed-state inactivation in voltage-gated ion channels. J. Physiol. 2011;589: 461–479. 10.1113/jphysiol.2010.191965 21098008 PMC3055536

[32] Klemic KG, Shieh CC, Kirsch GE, Jones SW. Inactivation of Kv2.1 potassium channels. Biophys. J. 1998;74: 1779–1789. 9545040 10.1016/S0006-3495(98)77888-9 PMC1299522

[33] Ader C, Schneider R, Hornig S, Velisetty P, Vardanyan V, Giller K, et al Coupling of activation and inactivation gate in a K+-channel: potassium and ligand sensitivity. EMBO J. 2009;28: 2825–2834. 10.1038/emboj.2009.218 19661921 PMC2750023

[34] Kerschensteiner D, Stocker M. Heteromeric assembly of Kv2.1 with Kv9.3: effect on the state dependence of inactivation. Biophys. J. 1999;77: 248–257. 10388754 10.1016/S0006-3495(99)76886-4 PMC1300326

[35] Madeja M, Steffen W, Mesic I, Garic B, Zhorov BS. Overlapping binding sites of structurally different antiarrhythmics flecainide and propafenone in the subunit interface of potassium channel Kv2.1. J. Biol. Chem. 2010;285: 33898–33905. 10.1074/jbc.M110.159897 20709754 PMC2962489

[36] Imbrici P, Grottesi A, D'Adamo MC, Mannucci R, Tucker SJ, Pessia M. Contribution of the central hydrophobic residue in the PXP motif of voltage-dependent K+ channels to S6 flexibility and gating properties. Channels (Austin) 2009;3: 39–45. 19202350 10.4161/chan.3.1.7548

[37] Robbins J. KCNQ potassium channels: physiology, pathophysiology, and pharmacology. Pharmacol. Ther. 2001;90: 1–19. 11448722 10.1016/s0163-7258(01)00116-4

[38] Thorneloe KS, Nelson MT. Properties and molecular basis of the mouse urinary bladder voltage-gated K^+^ current. J. Physiol. 2003;549: 65–74. 12679374 10.1113/jphysiol.2003.039859 PMC2342925

[39] Koh SD, Ward SM, Dick GM, Epperson A, Bonner HP, Sanders KM, et al Contribution of delayed rectifier potassium currents to the electrical activity of murine colonic smooth muscle. J. Physiol. 1999;515 (Pt 2): 475–487. 10050014 10.1111/j.1469-7793.1999.475ac.x PMC2269159

[40] Hristov KL, Chen M, Soder RP, Parajuli SP, Cheng Q, Kellett WF, et al Kv2.1 and electrically silent Kv channel subunits control excitability and contractility of guinea pig detrusor smooth muscle. Am. J. Physiol. Cell. Physiol. 2012;302: C360–C372. 10.1152/ajpcell.00303.2010 21998137 PMC3328844

[41] Zhu XR, Netzer R, Bohlke K, Liu Q, Pongs O. Structural and functional characterization of Kv6.2 a new gamma-subunit of voltage-gated potassium channel. Receptors and Channels 1999;6: 337–350. 10551266

[42] Cheng YM, Azer J, Niven CM, Mafi P, Allard CR, Qi J, et al Molecular determinants of u-type inactivation in kv2.1 channels. Biophys. J. 2011;101: 651–661. 10.1016/j.bpj.2011.06.025 21806933 PMC3145267

[43] Bahring R, Barghaan J, Westermeier R, Wollberg J. Voltage sensor inactivation in potassium channels. Front. Pharmacol. 2012;3: 100 10.3389/fphar.2012.00100 22654758 PMC3358694

[44] Kurata HT, Doerksen KW, Eldstrom JR, Rezazadeh S, Fedida D. Separation of P/C- and U-type inactivation pathways in Kv1.5 potassium channels. J. Physiol. 2005;568: 31–46. 16020465 10.1113/jphysiol.2005.087148 PMC1474772

[45] Claydon TW, Vaid M, Rezazadeh S, Kehl SJ, Fedida D. 4-aminopyridine prevents the conformational changes associated with p/c-type inactivation in Shaker channels. J. Pharmacol. Exp. Ther. 2007;320: 162–172. 17015639 10.1124/jpet.106.110411

[46] Barghaan J, Bahring R. Dynamic coupling of voltage sensor and gate involved in closed-state inactivation of Kv4.2 channels. J. Gen. Physiol. 2009;133: 205–224. 10.1085/jgp.200810073 19171772 PMC2638201

[47] Shin KS, Maertens C, Proenza C, Rothberg BS, Yellen G. Inactivation in HCN channels results from reclosure of the activation gate; desensitization to voltage. Neuron 2004;41: 737–744. 15003173 10.1016/s0896-6273(04)00083-2

[48] Decher N, Chen J, Sanguinetti MC. Voltage-dependent gating of hyperpolarization-activated, cyclic nucleotide-gated pacemaker channels: Molecular coupling between the S4-S5 and C-linkers. J. Biol. Chem. 2004;279: 13859–13865. 14726518 10.1074/jbc.M313704200

[49] Dougherty K, De Santiago-Castillo JA, Covarrubias M. Gating charge immobilization in Kv4.2 channels: the basis of closed-state inactivation. J. Gen. Physiol. 2008;131: 257–273. 10.1085/jgp.200709938 18299396 PMC2248721

[50] Cuello LG, Jogini V, Cortes DM, Pan AC, Gagnon DG, Dalmas O, et al Structural basis for the coupling between activation and inactivation gates in K(+) channels. Nature 2010;466: 272–275. 10.1038/nature09136 20613845 PMC3033755

[51] Cuello LG, Jogini V, Cortes DM, Perozo E. Structural mechanism of C-type inactivation in K(+) channels. Nature 2010;466: 203–208. 10.1038/nature09153 20613835 PMC3033749

[52] Pan AC, Cuello LG, Perozo E, Roux B. Thermodynamic coupling between activation and inactivation gating in potassium channels revealed by free energy molecular dynamics simulations. J. Gen. Physiol. 2011;138: 571–580. 10.1085/jgp.201110670 22124115 PMC3226968

[53] Yang YS, Yan YY, Sigworth FJ. How does the W434F mutation block current in Shaker potassium channels. J. Gen. Physiol. 1997;109: 779–789. 9222903 10.1085/jgp.109.6.779 PMC2217041

[54] Pless SA, Galpin JD, Niciforovic AP, Kurata HT, Ahern CA. Hydrogen bonds as molecular timers for slow inactivation in voltage-gated potassium channels. Elife 2013;2: e01289 10.7554/eLife.01289 24327560 PMC3852034

[55] Bocksteins E, Labro AJ, Snyders DJ, Mohapatra DP. The electrically silent Kv6.4 subunit confers hyperpolarized gating charge movement in Kv2.1/Kv6.4 heterotetrameric channels. PLoS ONE 2012;7: e37143 10.1371/journal.pone.0037143 22615922 PMC3355112

[56] Jackson AC, Bean BP. State-dependent enhancement of subthreshold A-type potassium current by 4-aminopyridine in tuberomammillary nucleus neurons. J. Neurosci. 2007;27: 10785–10796. 17913912 10.1523/JNEUROSCI.0935-07.2007 PMC6672835

[57] Shieh CC, Klemic KG, Kirsch GE. Role of transmembrane segment S5 on gating of voltage-dependent K^+^ channels. J. Gen. Physiol. 1997;109: 767–778. 9222902 10.1085/jgp.109.6.767 PMC2217039

[58] Caballero NA, Melendez FJ, Nino A, Munoz-Caro C. Molecular docking study of the binding of aminopyridines within the K^+^ channel. J. Mol. Model. 2007;13: 579–586. 17340113 10.1007/s00894-007-0184-9

[59] Czirjak G, Toth ZE, Enyedi P. Characterization of the heteromeric potassium channel formed by Kv2.1 and the retinal subunit Kv8.2 in Xenopus oocytes. J. Neurophysiol. 2007;98: 1213–1222. 17652418 10.1152/jn.00493.2007

[60] Yuan XJ, Wang J, Juhaszova M, Golovina VA, Rubin LJ. Molecular basis and function of voltage-gated K+ channels in pulmonary arterial smooth muscle cells. Am. J. Physiol. 1998;274: L621–L635. 9575881 10.1152/ajplung.1998.274.4.L621

