# Supplementary figures and images for: Modulation of Closed−State Inactivation in Kv2.1/Kv6.4 Heterotetramers as Mechanism for 4−AP Induced Potentiation
Source: PLoS One. 2015 Oct 27;10(10):e0141349. doi: 10.1371/journal.pone.0141349 (PMC4623978; doi:10.1371/journal.pone.0141349)

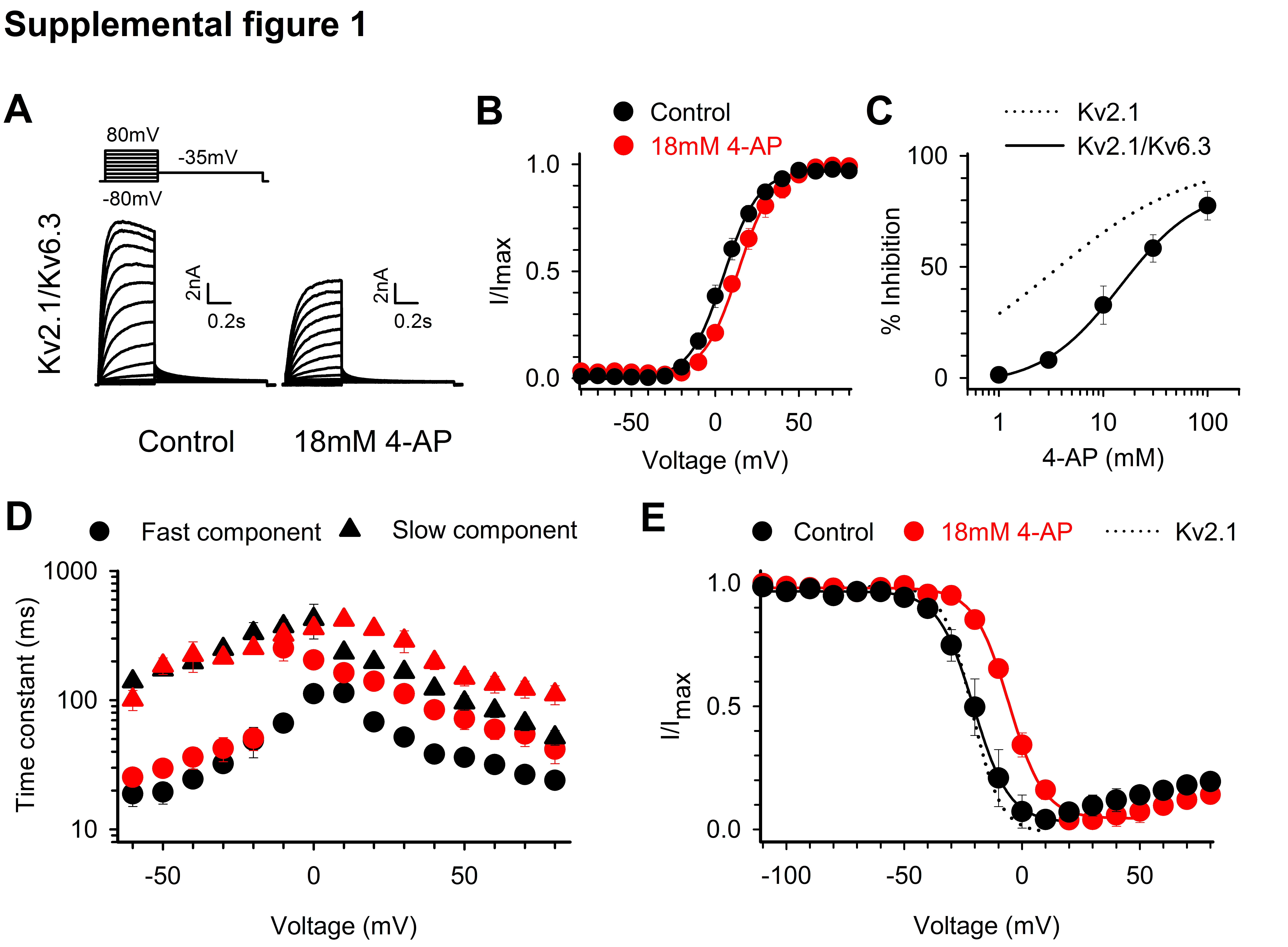

Supplement: S1 Fig — (A) Representative current recordings of Kv2.1/Kv6.3 before (left) and after application of 4-AP (right). 4-AP inhibited Kv2.1/Kv6.3 currents and modified the activation kinetics. Voltage protocol is shown on top. (B) Voltage dependence of activation with (red) and without 4-AP (black). 4-AP slightly shifted the activation curve towards depolarized potentials. (C) Concentration-dependence of the 4-AP inhibition. The blocking potency of 4-AP on Kv2.1/Kv6.3 is decreased in comparison to homotetrameric Kv2.1 channels (dotted line). The solid line represent a fit with the Hill equation with an IC50 of 15.5 ± 2.9 mM (n = 5). (D) Activation and deactivation kinetics of the Kv2.1/Kv6.3 heterotetramers. Time constants were fitted with a double exponential function and yielded a fast (circles) and slow (triangles) component. 4-AP (red) slowed both the fast and slow component of activation while the deactivation components remained unaffected. (E) The voltage dependence of inactivation before (black) and after 4-AP application (red). Kv6.3 does not affect the inactivation properties significantly as the inactivation curve overlaps completely with that of homotetrameric Kv2.1 channels (dotted line). 4-AP shifted the inactivation curve towards depolarized potentials and the extent of the shift was comparable to the shift of the activation curve. (TIF) [file pone.0141349.s001.tif]

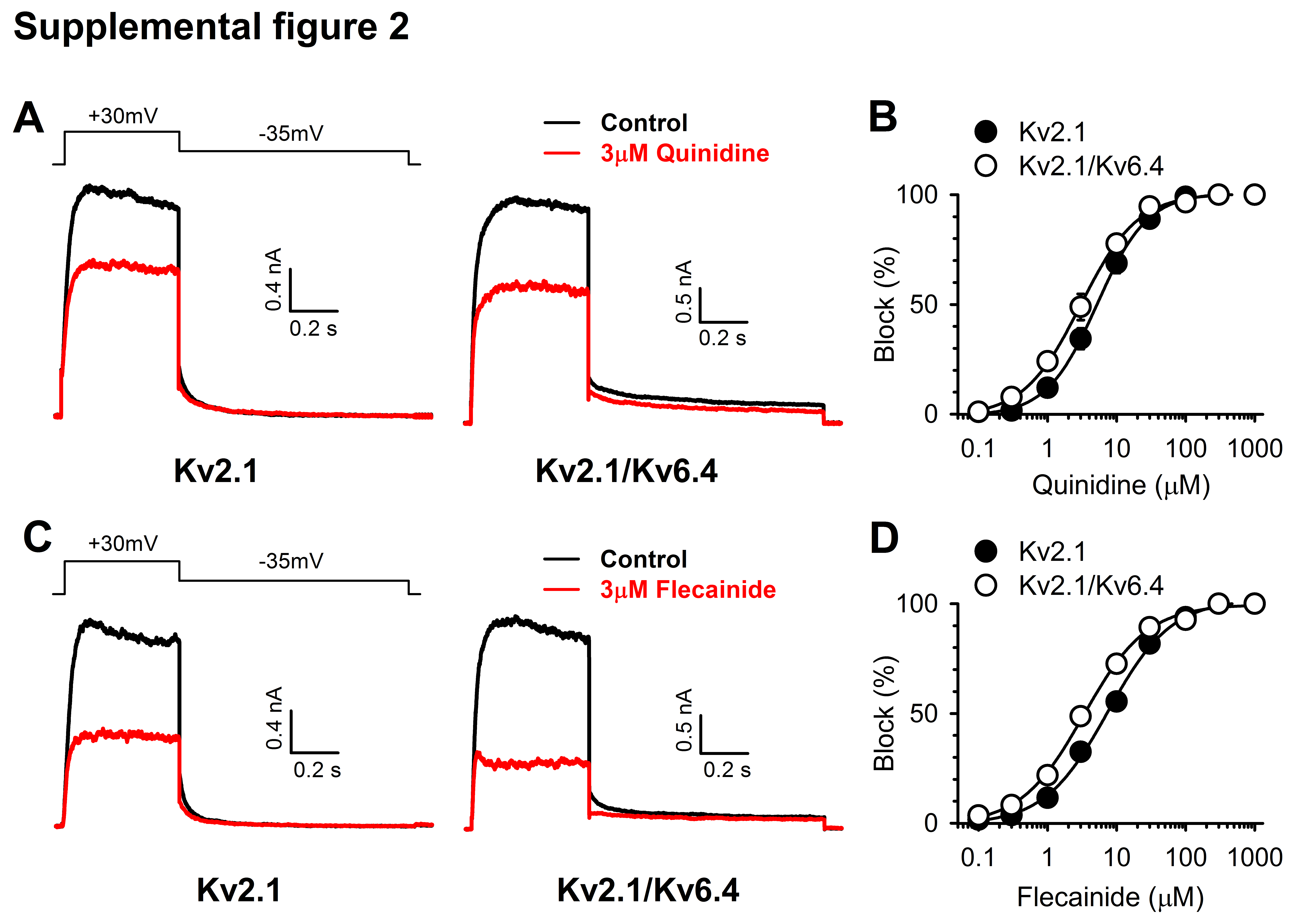

Supplement: S2 Fig — (A) Representative current recording before (black trace) and after 3 μM quinidine (red traces) of Kv2.1 (left) and Kv2.1/Kv6.4 channels (right). (B) Concentration dependence for inhibition by quinidine of Kv2.1 (filled circles) and Kv2.1/Kv6.4 (open circles) channels. Concentration−effect curves were obtained by plotting the normalized amount of block in function of the applied concentration and fitted with the Hill equation as described in the material and method section. Note the leftward shift of the Kv2.1/Kv6.4 concentration−effect curve compared to that of Kv2.1 homotetramers which corresponds to a twofold difference in IC50 value. (C) Effect of flecainide on Kv2.1 (left) and Kv2.1/Kv6.4 (right) channels. (D) Concentration−effect curves for flecainide inhibition of Kv2.1 (filled circles) and Kv2.1/Kv6.4 (open circles) channels obtained like the quinidine concentration−effect curve in panel B. As for quinidine, the heteromeric Kv2.1/Kv6.4 channels are more sensitive to flecainide. (TIF) [file pone.0141349.s002.tif]

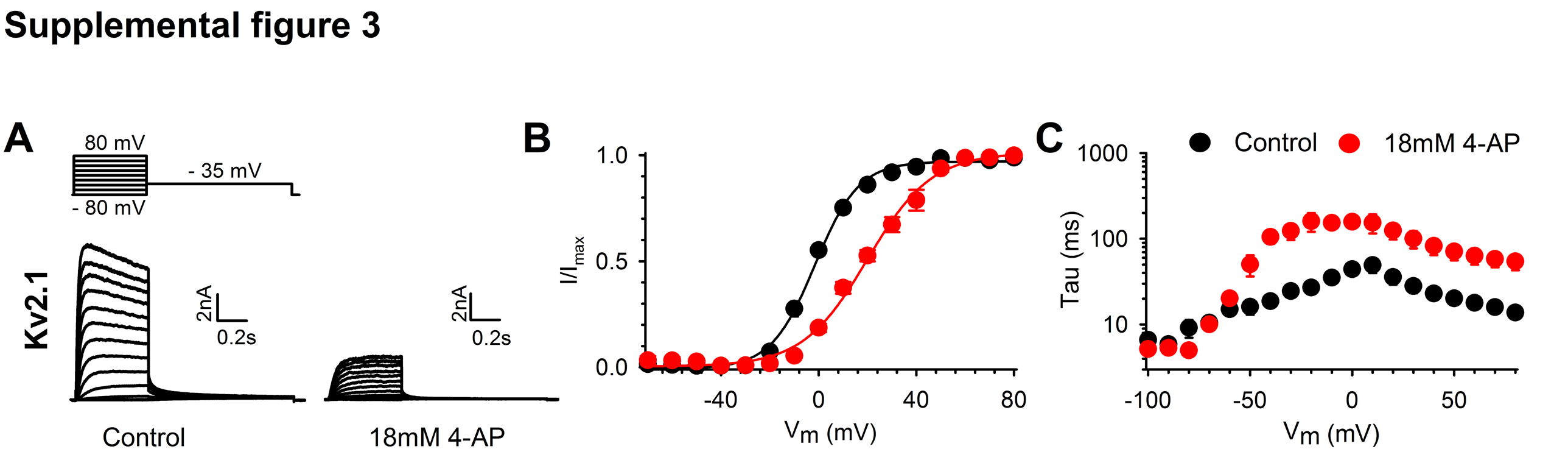

Supplement: S3 Fig — (A) Typical Kv2.1 current traces without (left) and with (right) 18 mM 4−AP revealing strong current inhibition and changes in activation kinetics. The pulse protocol is shown above the current traces. (B) Voltage dependence of activation for Kv2.1 obtained like for the WT Kv2.1/Kv6.4 heterotetramers in Fig 3. (C) Activation and deactivation kinetics of Kv2.1. Time constants were obtained with a single exponential function as previously described for the Kv2.1/Kv6.4_S6c_Kv9.3 chimera in Fig 6. Kv2.1 kinetics are shown as black circles and as red circles when 4−AP is present. (TIF) [file pone.0141349.s003.tif]

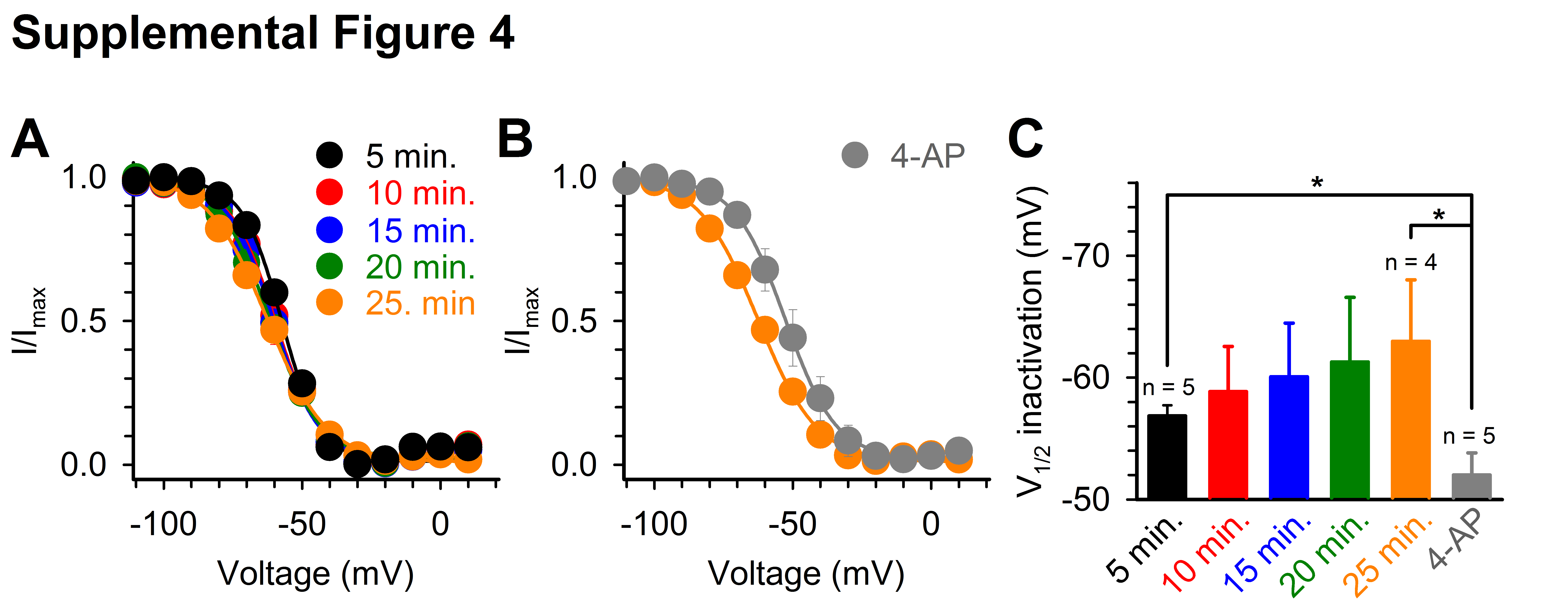

Supplement: S4 Fig — (A) Voltage dependence of inactivation, recorded as a function of time. Five inactivation protocols were recorded over a time span of 25 minutes and evaluated for a potential drift of the V1/2 of inactivation. A slight hyperpolarizing drift of the inactivation curve can be observed. (B) Comparison of the voltage dependence of inactivation before and after 4−AP application. After recording 5 repetitive inactivation protocols, 4−AP still shifted the inactivation curve towards depolarized potentials. (C) Bar chart showing the mean V1/2 of inactivation and S.E.M. at the different time intervals. 4-AP still shifted the V1/2 of inactivation significantly (* represent statistical significance) towards depolarized potentials. Colored dots/bars represent inactivation recorded, after obtaining a ‘whole cell’ patch configuration, at: 5 (black), 10 (red), 15 (blue), 20 (green) and 25 (orange) minutes. 4-AP data is shown in grey. (TIF) [file pone.0141349.s004.tif]

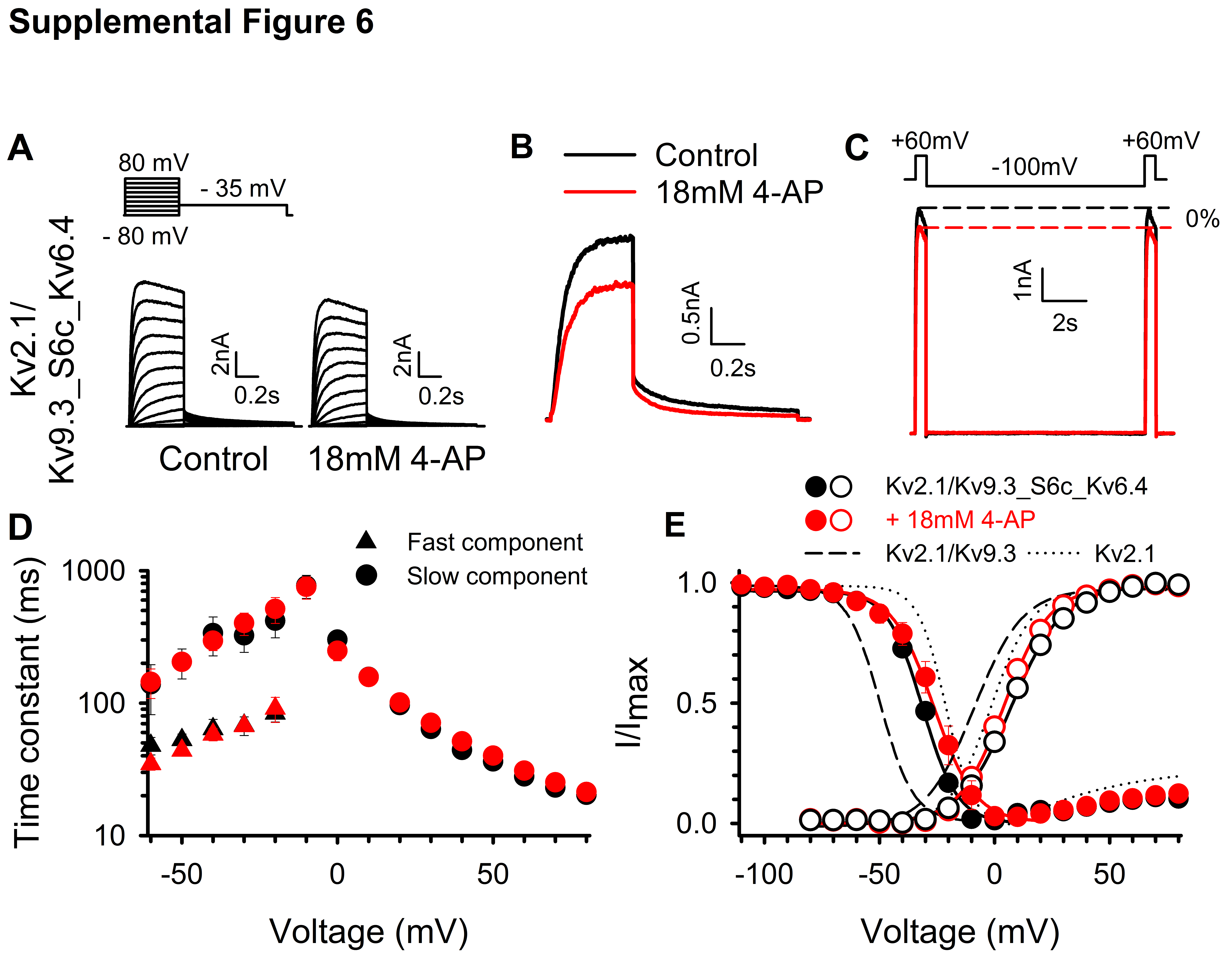

Supplement: S6 Fig — (A) Typical current traces for the Kv2.1/Kv9.3_S6c_Kv6.4 chimera without (left) and with 4−AP (right). The pulse protocol is shown on top. (B) 4−AP (red trace) slightly inhibited Kv2.1/Kv9.3_S6c_Kv6.4 currents (17 ± 3%, n = 6) comparable to the 12 ± 1% inhibition of WT Kv2.1/Kv9.3 currents seen in Fig 2. Control trace shown in black. (C) The twin pulse protocol from Fig 4E illustrates that the prominent closed−state inactivation was no longer present in Kv2.1/Kv9.3_S6c_Kv6.4 heterotetramers, seen as equal peak amplitudes at the two test pulses P1 and P2. 4−AP inhibited peak current amplitudes at P1 and P2 to a similar extent. (D) Activation and deactivation kinetics of the Kv2.1/Kv9.3_S6c_Kv6.4 heterotetramers (black circles). Time constants of activation were obtained with a single exponential function. Similar to the Kv2.1/Kv6.4_S6c_Kv9.3 chimera in Fig 6, the Kv2.1/Kv9.3_S6c_Kv6.4 chimera lacked a pronounced slow component present in Kv2/Kv6.4 heterotetramers (Fig 3C). (E) Voltage dependence of activation (open symbols) and inactivation (closed symbols) of the Kv2.1/Kv9.3_S6c_Kv6.4 chimera. Both were shifted towards depolarized potentials compared to the WT Kv2.1/Kv9.3 inactivation (dashed line), resulting in a loss of the pronounced closed−state inactivation process. 4−AP (red symbols) had no significant effect on the gating parameters. For comparison, homomeric Kv2.1 (dotted line) before 4−AP application is included. (TIF) [file pone.0141349.s006.tif]
